# Structural Basis of Human Kinetochore-Microtubule Coupling by the Ndc80 and Ska Complexes

**DOI:** 10.64898/2025.12.06.692780

**Published:** 2025-12-07

**Authors:** Ju Zhou, Yuanchang Zhao, Ahmet Yildiz, Eva Nogales

## Abstract

The outer kinetochore (KT) physically links chromosomes to dynamic microtubule (MT) plus ends, coupling to both polymerizing and depolymerizing tips to support chromosome movements while maintaining robust attachment. In human cells, the Ska complex is thought to function analogously to the yeast Dam1 complex and to cooperate with Ndc80 at the outer KT to ensure stable KT-MT interactions. However, the molecular basis for this cooperation remains poorly understood. We have obtained structures of human Ska and Ndc80 complexes simultaneously bound to MTs, showing how Ska interacts with MT across several tubulin dimers. Ndc80 and Ska complexes engage with each other across adjacent protofilaments “sandwiching” the α-tubulin C-terminal tail in the process. We also identify an anchoring interaction between a distinct bending point within the Ndc80 coiled-coil and a tethering helix and nearby phosphorylation sites (T358/T360) in SKA3. Our findings shed light on how human outer KT components collaboratively engage dynamic MT ends to contribute to robust KT-MT attachment and fidelity during chromosome segregation in mitosis.

## Full Text Availability

The license terms selected by the author(s) for this preprint version do not permit archiving in PMC. The full text is available from the preprint server.

