# Structural Basis of Human Kinetochore-Microtubule Coupling by the Ndc80 and Ska Complexes

**DOI:** 10.64898/2025.12.06.692780

**Published:** 2026-06-25

**Authors:** Ju Zhou, Ipsita Saha, Valerie Siahaan, Yuanchang Zhao, Carsten Janke, Ahmet Yildiz, Eva Nogales

**Affiliations:** 1 California Institute for Quantitative Biosciences (QB3), University of California, Berkeley, Berkeley, CA, USA; 2 Physics Department, University of California, Berkeley, CA, USA; 3 Department of Molecular and Cell Biology, University of California, Berkeley, CA, USA; 4 Institut Curie, Université PSL, CNRS UMR3348, INSERM U1368, Orsay, France; 5 Université Paris-Saclay, CNRS UMR3348, INSERM U1368, Orsay, France; 6 Molecular Biophysics and Integrative Bioimaging Division, Lawrence Berkeley National Laboratory, Berkeley, CA, USA; 7 Biophysics Graduate Group, University of California, Berkeley, CA, USA; 8 Howard Hughes Medical Institute, University of California, Berkeley, Berkeley, CA, USA

## Abstract

Faithful chromosome segregation depends on kinetochore-microtubule attachments that must be load-bearing, dynamic, and tightly regulated. In human cells, the Ska and Ndc80 complexes (SkaC and Ndc80C) cooperate to sustain these attachments, but how they are organized on microtubules and coupled to end dynamics remains unclear. Here, our cryo-EM structure shows that Ndc80C and SkaC form a coupled assembly across neighboring protofilaments. A phosphorylation-sensitive SKA3 tether binds a defined kink in the Ndc80 coiled-coil, positioning SKA1 beside Ndc80C. These two complexes jointly sandwich the α-tubulin tail and sense its polyglutamylation state. SkaC recognizes the extended microtubule lattice and, through the SKA3 tether, confers this selectivity onto Ndc80C, enabling Ndc80C tip tracking while strengthening microtubule stabilization. Together, our findings define the structural basis of Ndc80C-SkaC cooperation and uncover a phosphorylation-regulated, polyglutamylation-sensitive, and lattice-state-dependent mechanism for robust and regulated KT-MT coupling during chromosome segregation in mitosis.

## INTRODUCTION

During cell division, accurate segregation of chromosomes requires kinetochore-microtubule (KT-MT) attachments that are both mechanically robust and dynamically adaptable^1–3^. These attachments must sustain force while remaining coupled to MT plus ends as they grow and shorten during chromosome congression and segregation^4–6^. Failure to maintain this balance can produce erroneous or hyperstable attachments that evade error correction, leading to chromosome mis-segregation, aneuploidy, and chromosome instability associated with cancer and developmental disease^7–10^.

At the outer KT, the KNL1, Mis12, and Ndc80 complexes form the conserved KMN network that links chromosomes to spindle MTs^2,11^. The Ndc80C is the principal MT-binding and force-coupling element of this network^4,5^. Human Ndc80C is an elongated heterotetramer composed of NDC80, NUF2, SPC24, and SPC25, with the NDC80-NUF2 calponin-homology domains binding MTs and the SPC24-SPC25 end connecting to inner KT receptors^12–16^. Although Ndc80C can bind cooperatively along MTs, how this core attachment complex is converted into a regulated assembly that remains coupled to dynamic MT plus ends remains unresolved^2,5,14^.

Eukaryotic KTs share the challenge of maintaining persistent attachment to dynamic MT ends, but the molecular solutions to this problem have diverged. In budding yeast, Dam1C forms oligomeric MT-associated assemblies and couples to Ndc80C through defined interfaces^17–22^. Metazoan KTs lack Dam1C and instead rely on SkaC to reinforce mature KT-MT attachments^23–25^. SkaC accumulates at KTs as MT attachments mature^23,26^, promotes stable end-on attachment^27,28^, prevents force-dependent detachment^26^, and supports MT-end tracking^24,29–31^. SkaC is organized as a W-shaped homodimer of SKA1-SKA2-SKA3 heterotrimers^32,33^. SKA1 contains an MT-binding domain (MTBD), whereas the extended C-terminal region of SKA3 interacts with Ndc80C in a manner enhanced by cyclin-dependent kinase phosphorylation^30,31,33,34^. While these properties establish SkaC as a key regulator of mature KT-MT, the molecular architecture by which SkaC is physically coupled to Ndc80C on the MT surface, and how this coupling contributes to MT dynamics and supports persistent plus-end tracking, has remained incompletely defined^30,31,35^.

How SkaC is positioned with respect to Ndc80C on MTs remains unresolved^24–26,30,31,35^. Previous studies have implicated different Ndc80C regions in SkaC binding, including the NDC80 N-terminal tail^35,36^, the internal loop^28,34,37^, and coiled-coil regions^30,33^. Previous studies also proposed distinct modes of SkaC-MT engagement, including single-PF binding^35^, multi-PF or multi-orientation binding^32,33^, and larger oligomeric or multivalent assemblie^38^. Therefore, the molecular architecture that organizes human Ndc80C and SkaC into a regulated, plus-end-coupled attachment is still unclear.

By integrating cryo-electron microscopy (cryo-EM) with biochemical assays and single-molecule measurements on dynamic MTs, we define the architecture of the human Ndc80C-SkaC assembly on the MT surface and assess how this organization controls PTM-sensitive MT engagement, lattice-state selectivity, regulation of MT dynamics, and plus-end coupling. Our findings reveal that SkaC is not simply an accessory MT binder, but a regulated partner that, through a SKA3-mediated interface, couples Ndc80C to the structural state and dynamics of MT ends. This mechanism provides a framework for how human KTs maintain robust yet adaptable chromosome attachments.

## RESULTS

### Ndc80C and SkaC simultaneously bind adjacent protofilaments

We purified recombinant full-length human SkaC and Ndc80C for cryo-EM analysis (Extended Data Fig. 1) and used a phosphomimetic SkaC-6D carrying aspartate substitutions to mimic phosphorylation that has been shown to enhance interaction with Ndc80C^39^ Extended Data Fig. 1a). In samples containing Ndc80C alone, MTs were seen uniformly coated by Ndc80C, with sharp edges corresponding to the coiled-coil region parallel to the MT axis of ~660 Å outer diameter (Extended Data Fig. 2a). In the presence of SkaC-6D, the Ndc80C coiled-coil regions appeared less ordered, indicating a distinct MT decoration pattern (Extended Data Fig. 2b).

We determined the average structure of Ndc80C and SkaC co-bound to MTs at resolutions ranging from 2.8 Å (tubulin) to 6 Å (higher-radius, visible segment of Ndc80C) (Extended Data Figs. 2–4; Supplementary Video 1; Supplementary Table 1). Ordered segments of the complexes span a diameter of approximately 550 Å (Extended Data Fig. 5a), whereas density beyond was not resolved, reflecting its conformational flexibility.

Consistent with prior observations^14,40^, Ndc80C binds along a PF with tubulin-monomer periodicity (Extended Data Fig. 2c,d). By contrast, SkaC binds with a tubulin dimer repeat at the longitudinal interdimer interface between adjacent PFs (Extended Data Figs. 2d and 5a,b). In the averaged structure, the SKA1-MTBD density partially overlaps with that of the NDC80-CHD (Extended Data Fig. 5a,b and Supplementary Video 1), revealing a steric clash and indicating that particles with different Ndc80C- or SkaC-bound registries were mixed during averaging. 3D classification at the single tubulin dimer level confirmed that Ndc80C and SkaC do not simultaneously bind the same tubulin (Extended Data Fig. 2d, left panel).

**Fig. 1: |:**
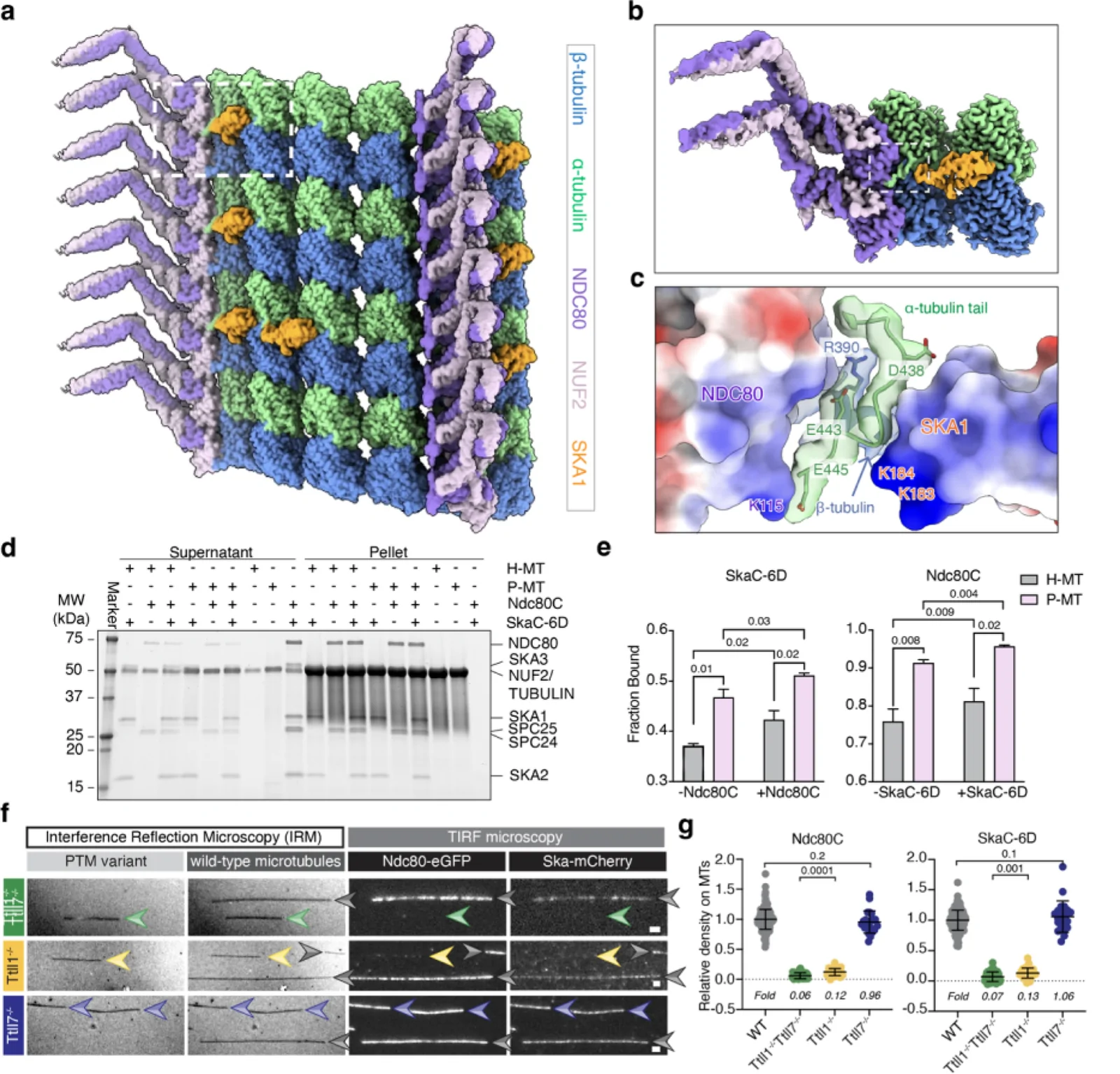
Structural and biochemical basis of the MT-bound Ndc80C-SkaC “sandwich” conformation. **a,** Composite cryo-EM map illustrating the different arrangements of Ndc80C and SkaC on the MT surface identified by 3D classification (“SkaC” refers to the SkaC-6D construct in this study unless otherwise indicated.). The white dashed box highlights the class in which Ndc80C and SkaC are simultaneously bound to two adjacent PFs. Maps were sharpened using DeepEMhancer for visualization. **b,** Enlarged view of the boxed region in (**a**). **c,** Close-up view of the “sandwich” interface formed by the NDC80 CHD, the α-CTT, and the SKA1-MTBD. NDC80 and SKA1 are shown as electrostatic surfaces. Key residues involved in α-CTT stabilization are indicated. A segment of β-tubulin is also shown, with the R390 side chain displayed as sticks. **d,** SDS-PAGE analysis of MT-pelleting assays showing binding of Ndc80C and SkaC to HeLa-MTs and porcine brain MTs. **e,** Quantification of the experiments shown in (**d**) for SkaC (left) and Ndc80C (right), with the pelleted SkaC or Ndc80C signal normalized to the corresponding pelleted MT signal (mean ± SD; three independent experiments). SkaC binding was quantified using the SKA2 band, and Ndc80C binding was quantified using the NDC80 band. P-values are calculated by a two-tailed t-test. **f,** GMPCPP MTs assembled from knockout mouse brain tubulin (indicated by colored arrowheads) or wild-type (WT) mouse brain tubulin (grey arrowheads) were sequentially immobilized in the measurement chamber and visualized using IRM (Interference Reflection Microscopy). 4 nM Ndc80C-eGFP and 4 nM SkaC-mCherry were added to the chamber (columns 3 and 4, respectively) and imaged using TIRF microscopy. Scalebars: 2 μm. **g,** Quantitative analysis of relative Ndc80C (left) or SkaC (right) density on WT and PTM-variant MTs. Fluorescence intensities of Ndc80C or SkaC on PTM-variant MTs were normalized to those on WT MTs. Data are presented as mean ± SD. (n=169, 44, 37, 22 MTs in 9, 3, 3, 3 independent experiments, respectively). Statistical significance was determined using two-tailed t-test; p-value and fold differences relative to WT displayed in the plot.

We next asked whether SkaC and Ndc80C can bind adjacent tubulin dimers along the same PF. 3D classification on two longitudinal dimers identified either no complex bound, Ndc80C bound consecutively to four tubulin monomers, or SkaC bound at the interdimer junction (Extended Data Fig. 2d, middle panel). Thus, SkaC and Ndc80C are unlikely to bind to longitudinally adjacent tubulin dimers, probably due to cooperative Ndc80C binding along a PF. In contrast, classification of two lateral dimers revealed a class in which SkaC and Ndc80C are simultaneously engaging two adjacent PFs (Extended Data Fig. 2d, right panel, and Extended Data Fig. 2e). This result indicates that at the human outer KT, Ndc80C clustering along one PF promotes SkaC recruitment to the adjacent PF (Fig. 1a,b).

### Ndc80C and SkaC sandwich the α-tubulin tail

Our analysis revealed a previously uncharacterized mode of cooperative tubulin engagement of the two KT complexes: the C-terminal tail of α-tubulin (α-CTT, residues 438–445) is “sandwiched” between the NDC80 CHD and the SKA1 MTBD across two adjacent PFs (Fig. 1b,c). Tubulin tails are negatively charged peptides that often cannot be resolved by cryo-EM due to their inherent flexibility and sequence variation among α-tubulin isoforms. However, α-CTT becomes ordered when sandwiched between SkaC and Ndc80C, and D438, E443, and E445 are conserved (Extended Data Fig. 5c), enabling confident modeling of the α-CTT density up to E445 (Fig. 1c).

The NDC80 and SKA1 interfaces that engage the αCTT are strongly positively charged (Fig. 1c). NDC80 K115 and β-tubulin R390 form salt bridges with α-tubulin E445 and E443, respectively. On the other side of the interface, the α-CTT is engaged by SKA1 K183 and K184. Our structure indicates that αCTT stabilizes Ndc80C-SkaC interaction by mediating simultaneous interactions with both complexes. This is consistent with the NDC80 K115E mutation to impair KT-MT attachments and chromosome alignment^41^. The interactions we observed between SKA1 and α-CTT also provide an explanation for how SKA1 mutants that neutralize K183 and K184 weaken MT end tracking and increase chromosome alignment defects *in vivo*^42^ while reducing MT binding *in vitro*^33^.

When adjacent PFs are not co-occupied with Ndc80C on the left and SkaC on the right (when facing towards the MT plus-end), the α-CTT extends outward to interact with the N-terminal tail (NTT) of NDC80 in the adjacent PF (Extended Data Fig. 5d), as previously described^14,40^. We also observed stabilization of the β-tubulin CTT, up to residue E435, by interaction with the NDC80 CHD (Extended Data Fig. 5e). These findings further suggest that tubulin isotype-specific CTT sequences and their PTMs can regulate KT-MT interactions.

### Tubulin tails and polyglutamylation modulate cooperative Ndc80C-SkaC binding

Previous studies have shown that Ndc80C binding to MTs depends strongly on the tubulin CTTs^12,33^, whereas the reported effects on SkaC binding have been less consistent^33,43^. To quantify the functional significance of the tubulin tails in Ndc80C and SkaC binding to MTs, we removed the tubulin CTTs by subtilisin treatment and assessed MT binding by pelleting assays. Subtilisin treatment markedly reduced binding of Ndc80C alone, SkaC alone, or co-binding of the two complexes together (Extended Data Fig. 5f). These results indicate that the tubulin tails contribute to both the individual association of Ndc80C and SkaC with MTs^12,43^ and their co-engagement with the MT surface.

α-tubulin E445, the most C-terminal residue seen in our structure, is a preferential site of polyglutamylation, a post-translational modification (PTM) that regulates MT dynamics and chromosome segregation^44–47^ (Fig. 1c). To test whether tubulin PTMs could directly modulate Ndc80C and SkaC binding, we first compared their binding to HeLa-MTs which carry low levels of tubulin tail modifications^48^, and porcine brain MTs, which are highly post-translationally modified^49^. Because α- and β-tubulin sequences are highly conserved between human and porcine tubulin (Extended Data Fig. 6a,b), this comparison allowed us to assess the contribution of the tubulin modification state. In MT pelleting assays, Ndc80C alone, SkaC alone, and the two complexes together, all exhibited weaker binding to HeLa MTs than to porcine brain MTs (Fig. 1d,e). Consistently, TIRF-based titration experiments showed reduced recruitment of Ndc80C and SkaC to HeLa MTs, particularly at lower concentrations of these complexes (Extended Data Fig. 7a,b), supporting that some of the tubulin PTMs that differentiate these two types of tubulin can modulate the association of the outer-KT Ndc80C and SkaC complexes with the MT lattice.

We next focused most specifically on polyglutamylation, the modification that should impart the largest effect on MT binding by both Ndc80C and SkaC based on our structure. Using MTs from mutant mouse brain lacking defined tubulin-polyglutamylation enzymes, we found that absence of both TTLL1 and TTLL7, or TTLL1 alone, strongly reduced binding to MTs by both Ndc80C and SkaC. In contrast, MTs from TTLL7-KO mice retained near wild-type levels of Ndc80C and SkaC binding (Fig. 1f,g). Because TTLL1 preferentially modifies α-tubulin, whereas TTLL7 primarily modifies β-tubulin^50,51^, these results indicate that α-tubulin polyglutamylation promotes Ndc80C-SkaC engagement with MTs.

Thus, our data identify the α-CTT as a key element in an interaction hub for the human outer KT that can be regulated via polyglutamylation. Sandwiching of the α-CTT between Ndc80C and SKA1 provides a molecular explanation for how tubulin-tail modifications, particularly α-tubulin polyglutamylation, can tune the cooperative recruitment of Ndc80C and SkaC to the MT lattice.

### Molecular interactions at the MT-Ndc80C interface

**Fig. 2: |:**
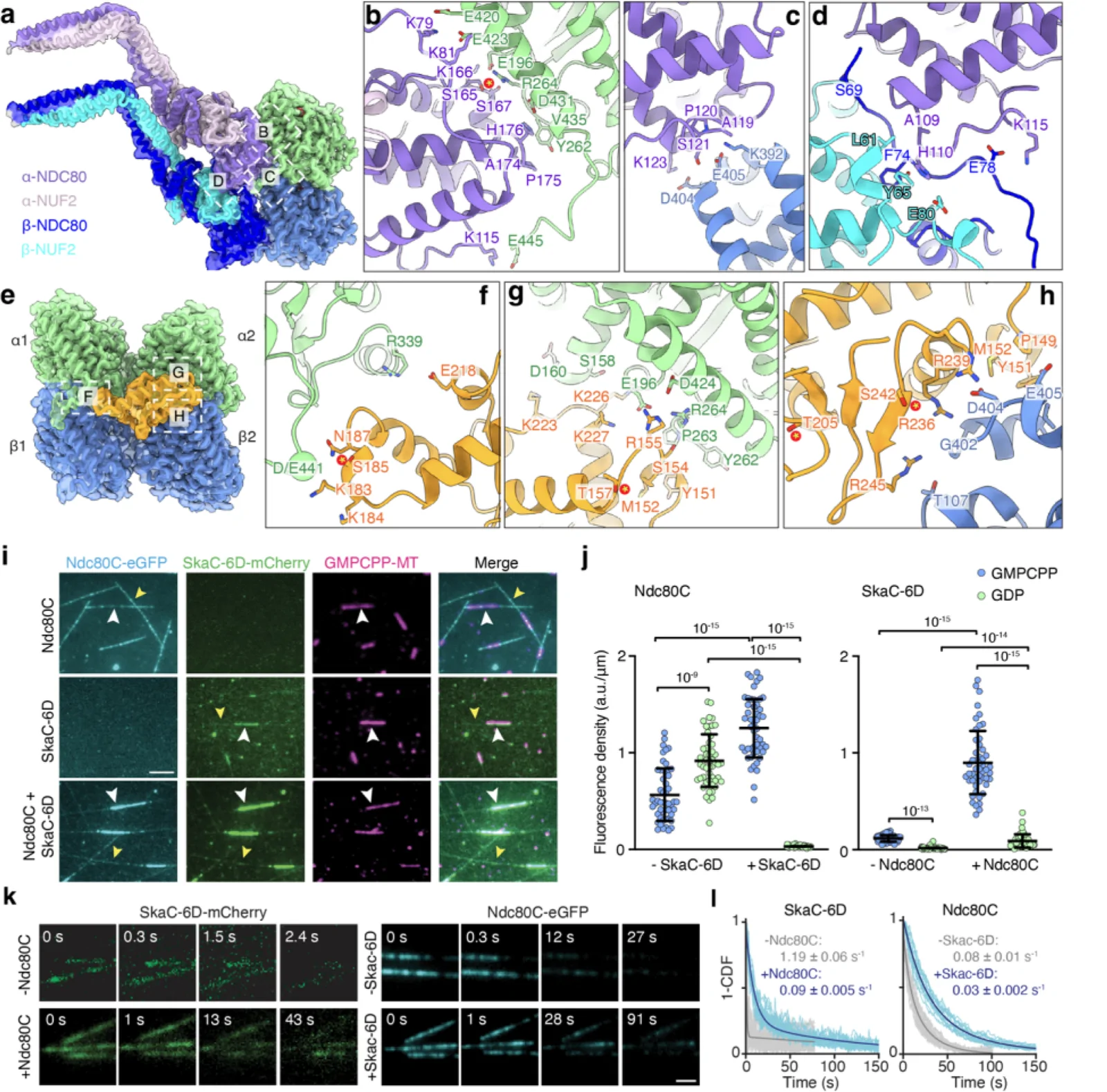
Distinct MT interactions shape cooperative and lattice-selective Ndc80C-SkaC binding. **a,** Cryo-EM map with fitted models showing two adjacent Ndc80Cs, denoted as α-Ndc80C and β-Ndc80C, bound to a tubulin dimer. Regions shown in detail on the right are indicated. Maps were sharpened using DeepEMhancer for visualization (same for (**e**)). **b,** Close-up view of the interface between α-Ndc80C and α-tubulin. Key residues are labeled and shown as sticks, and phosphorylation sites are marked by asterisks (same for panels (**c-h**)). **c,** Close-up view of the interface between α-Ndc80C and β-tubulin. **d,** Interface between two adjacent Ndc80C. **e,** Cryo-EM map with fitted models showing the SKA1-MTBD (orange) bound at the interdimer interface between two adjacent PFs. For clarity, tubulin subunits are labeled α1, α2, β1, and β2. Regions shown in detail on the right are indicated. **f,** Close-up view of the interface between SKA1 and α1-tubulin. **g,** Close-up view of the interface between SKA1 and α2-tubulin. **h,** Close-up view of the interface between SKA1 and β2-tubulin. **i,** Representative images showing binding of Ndc80C-eGFP alone (50 nM), SkaC-6D-mCherry alone (100 nM), and Ndc80C-eGFP in the presence of SkaC-6D-mCherry on GMPCPP MT lattices (magenta) versus GDP lattices (unlabeled). White and yellow arrowheads indicate binding on the GMPCPP and GDP lattice, respectively. Images were individually contrast-adjusted to visualize weak binding regions. Scale bar: 5 μm. **j,** Integrated fluorescence density of Ndc80C and SkaC-6D under the indicated conditions. The center line and whiskers represent the mean ± SD. P values were calculated using a two-tailed t-test. N = 50 MTs were analyzed for each condition from two independent experiments. **k,** Representative images showing the localization of SkaC-6D-mCherry (green) and Ndc80C-eGFP (cyan) on GMPCPP MTs. Time stamps denote elapsed time after washout. Scale bar: 2 μm. **l,** The inverse cumulative distribution function (1-CDF) of SkaC-6D-mCherry (left) and Ndc80C-eGFP (right) intensity per length of an MT. Decay constants (±SD) were calculated from a fit to an exponential decay (solid curves, N = 20 MTs for each condition).

As previously shown at lower resolution^14,40^, alternating Ndc80Cs contact α- and β-tubulin along a PF (Fig. 2a). We denote the complexes that predominantly engage α-tubulin or β-tubulin as α-Ndc80C and β-Ndc80C, respectively (Fig. 2a). The α-Ndc80C interface with α-tubulin involves a combination of electrostatic, hydrogen-bonding, and hydrophobic contacts (Fig. 2b,c and Extended Data Fig. 8a–c). In particular, residues K115, K166, S165/S167, and H176 contribute to α-tubulin engagement, with NDC80 S165 notable as a reported NEK2 phosphorylation site^52–54^ (Fig. 2b and Supplementary Video 1). Additional hydrophobic contacts centered on A174, P175, and H176 further reinforce this interface (Fig. 2b and Extended Data Fig. 8c). By contrast, α-Ndc80C makes fewer contacts with β-tubulin (Fig. 2c and Extended Data Fig. 8d). β-Ndc80C engages tubulin in a closely analogous manner, consistent with conservation of the relevant contact surfaces in α- and β-tubulin (Extended Data Fig. 8e,f) and with the monomer-repeat binding pattern of Ndc80C on MTs.

### Cooperative binding of Ndc80C on the MT surface

As human Ndc80C binds to both tubulin monomers (Fig. 2a), it self-associates into clusters along a PF^14,40^. Direct inter-complex contacts are mediated in part by the NDC80 NTT, with β-NDC80 residues N68-K81 inserting into a groove formed between α-NDC80 and β-NUF2 (Fig. 2d and Extended Data Fig. 9a,b). This interface is stabilized by a hydrophobic patch centered on β-NDC80 F74, β-NUF2 Y65, and α-NDC80 H110, together with adjacent acidic residues involving β-NDC80 E78 and β-NUF2 E80 that contribute electrostatic complementarity. Notably, NDC80 S69, an Aurora A phosphorylation site^55^, faces the negatively charged surface of the adjacent complex (Extended Data Fig. 9c). The interfacial residues are highly conserved across vertebrates but diverge in invertebrates (Extended Data Fig. 9d), consistent with budding yeast Ndc80C binding MTs with tubulin-dimer periodicity^20^, rather than the monomer-repeat arrangement.

Lowering the contour threshold revealed additional density extending from a previously uncharacterized kink (NDC80^288–292^/NUF2^170–174^) in the coiled-coil to an interhelical loop in a neighboring Ndc80C (Extended Data Fig. 10a). This kink is distinct from the canonical hinge region (NDC80^359–362^/NUF2^244–247^) and is absent in budding yeast^16,20,56–59^ (Extended Data Fig. 10b). AlphaFold3 (AF3)^60^ predicts a straight and continuous coiled-coil from the kink to the hinge (Extended Data Fig. 10a). Beyond the hinge, the distal Ndc80C coiled-coils (NDC80^363–642^/NUF2^248–464^), including the loop, are predicted by AF3 to form bundles with a spacing of ~40 Å (Extended Data Fig. 10c), which matches both the tubulin repeat on MTs and the inter-Ndc80C spacing in our images (Extended Data Fig. 10d). Together, these features support a model in which a network of MT-stabilized contacts promotes cooperative Ndc80C clustering, thereby reinforcing robust KT-MT attachment.

### SkaC bridges tubulin interfaces on the MT lattice

Our cryo-EM map shows the SKA1 MTBD at a four-tubulin junction on the MT lattice, where it spans both the longitudinal interdimer interface along a PF, and the lateral interface between neighboring PFs (referred to as α1-, α2-, and β2-tubulins, Fig. 2e). The most extensive and well-defined contacts are formed with β2-tubulin, whereas the α1- and α2tubulin interfaces are less well resolved (Fig. 2f–h and Extended Data Fig. 11a–d), suggesting local conformational flexibility. Overall, the SKA1-MT interface is dominated by basic SKA1 residues that contact acidic tubulin surfaces, with additional local hydrophobic reinforcement (Extended Data Fig. 11e,f). At the α1-tubulin interface, K183 and K184 lie opposite acidic residues in the α1-tubulin CTT, whereas the Aurora B site S185^27,29,33^ is positioned nearby within the same contact region (Fig. 2f and Supplementary Video 1). The adjacent α2-tubulin interface contains a positively charged SKA1 patch, including K223, K226, K227, and R155, and is further stabilized by a hydrophobic contact centered on SKA1 Y151-M152 and α2-tubulin Y262-P263 (Fig. 2g and Extended Data Fig. 11e). Notably, the Aurora B site T157 also maps close to this interface.^27,33^ At the β2-tubulin interface, basic residues R236, R239, and R245 contribute additional polar contacts near the αβ-tubulin interdimer junction, whereas P149, Y151, and M152 provide hydrophobic reinforcement (Fig. 2h and Extended Data Fig. 11f). The Aurora B site S242^27,33^ lies within this interface, whereas T205^27,33^ is positioned more peripherally, suggesting that phosphorylation at these sites could directly or allosterically modulate SkaC-MT affinity. These structurally defined contact sites provide an explanation for previous mutational analyses that identified the SKA1 basic patches and Aurora B phospho-sites within the MTBD as key determinants of MT binding and mitotic function^27,29,33,42^.

The SKA1 MTBD is separated from the hetero-trimerization and homo-dimerization region of the SkaC by a ~40-residue unstructured segment (Extended Data Fig. 1a)^32,33^. Therefore, our reconstruction cannot define how the two MTBDs within a SkaC dimer are arranged relative to one another on the MT. When we mapped the SKA1 patches identified by cross-linking mass spectrometry (XL-MS) as being near tubulin onto our SKA1-MT structure, SKA1 K183/K184 are positioned >30 Å from β-tubulin, and K203/K206 lie >30 Å from α-tubulin^33^ (Extended Data Fig. 11g–i) and therefore do not contribute to the interaction of SKA1 with the MT (Fig. 2e–h). Instead, mutating these SKA1 residues was shown to abolish SKA1 binding to curved-tubulin^42^, suggesting that the SKA1 MTBD contains a distinct tubulin-interaction surface that recognizes curved tubulin.

### SkaC biases Ndc80C toward the extended MT lattice

Upon GTP hydrolysis following tubulin polymerization, conformational changes in GDP-bound tubulin give rise to a lattice compaction at the dimer interface between PFs^61^, whereas GTP caps at the MT ends adopt an extended conformation. The interaction of SKA1 across tubulin dimers suggests that engagement of SkaC with the MT may be sensitive to the nucleotide-dependent state of the lattice (Fig. 2e). To test this idea, we used single-molecule fluorescence imaging to examine the behavior of Ndc80C and SkaC on distinct MT lattices in vitro. We used GMPCPP-stabilized seeds, which mimic an extended, GTP-like lattice, to nucleate dynamic MTs, which themselves hydrolyze GTP and thus have a compacted GDP lattice^61^.

Consistent with previous observations^42^, SkaC alone exhibited weak diffusive binding along the GDP lattice, with a preference for the GMPCPP seeds (Fig. 2i,j). In the presence of Ndc80C, however, SkaC’s affinity for the MT increased significantly, and its preference for the GTP-like lattice became more pronounced (Fig. 2i,j). Ndc80C alone exhibited diffusive behavior along both GTP-like and GDP lattices, with a slight preference for the latter (Fig. 2i,j). Strikingly, in the presence of SkaC-6D, Ndc80C became selectively enriched on the GTP-like lattice (Fig. 2i,j). These observations reveal cooperative binding of SkaC and Ndc80C on MTs and indicate that SkaC directs Ndc80C toward GTP-like regions, promoting attachment to growing MT ends at the outer KT. Additionally, we measured dissociation kinetics of Ndc80C and SkaC from GMPCPP MTs after removing unbound complexes from the flow chamber (Fig. 2k,l). The relatively rapid dissociation of SkaC was slowed by more than an order of magnitude in the presence of Ndc80C. Similarly, SkaC reduced the dissociation rate of Ndc80C by 3-fold. Together, these data support a reciprocal interaction in which SkaC biases Ndc80C towards the GTP-like lattice, while Ndc80C stabilizes SkaC association with MTs.

### SKA3 interacts with a vertebrate-conserved kink in the Ndc80C coiled-coil

**Fig. 3: |:**
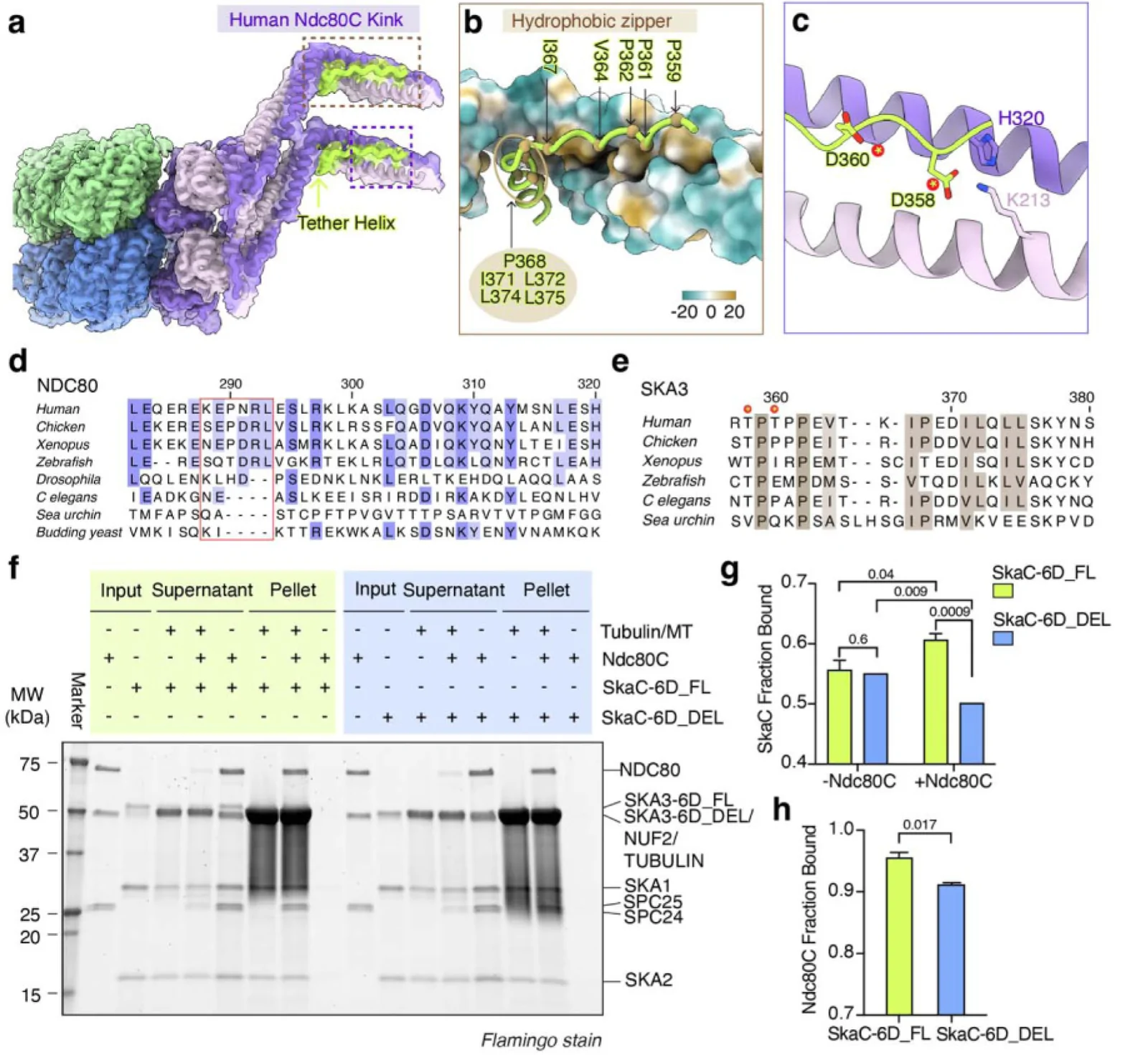
A phosphosite-containing SKA3 tether engages the Ndc80C kink. **a,** Cryo-EM density with fitted models showing the SKA3 tether helix and preceding region (residues 357–380) bound to the Ndc80C kink. Overview density is shown from the composite map, whereas the local SKA3-Ndc80C kink region density is shown from the Relion B-factor-sharpened map. **b,** Close-up view of the hydrophobic interface between the SKA3 segment and the Ndc80C kink. NDC80-NUF2 are shown as a hydrophobicity-colored surface, and SKA3 is shown as a ribbon with hydrophobic residues depicted as spheres. **c,** SKA3 residues T358 and T360 lie within the Ndc80C-binding interface and are represented here by the phosphomimetic substitutions D358 and D360 in the SkaC-6D construct used for EM studies. **d,** Sequence alignment of the Ndc80 kink and adjacent residues forming the SKA3-interacting interface shown in (**a**). Kink residues are boxed in red, and conserved interface residues are highlighted. Darker shading denotes higher sequence conservation. **e,** Sequence alignment of the SKA3 tether segment engaging the Ndc80 kink in (**a**) across the species in (**d**), excluding budding yeast. Hydrophobic residues at the interface positions are highlighted. **f,** Flamingo-stained SDS-PAGE analysis of MT-pelleting assays showing SkaC-6D_FL and SkaC-6D_DEL binding to MTs in the absence or presence of Ndc80C. **g,h,** Quantification of the MT binding ratios of SkaC (**g**) and Ndc80C (**h**) from the experiments shown in (**f**). SkaC binding was quantified using the SKA2 band, and Ndc80C binding was quantified using the NDC80 band. Data are mean ± SD from three independent experiments, analyzed by a two-tailed t-test.

Our structure shows the presence of a previously uncharacterized kink in the Ndc80C coiled-coil (NDC80^288–292^/NUF2^170–174^) that lies proximal to the canonical hinge (NDC80^359–362^/NUF^244–247^, Fig. 3a–d and Extended Data Fig. 10a,d). This kink is absent from the yeast Ndc80C (Extended Data Fig. 10b), and sequence conservation analysis suggests that it is more prominent in vertebrates (Fig. 3d). Interestingly, we observed additional density associated with this bending region, but it could not be directly assigned due to lower resolution (Fig. 3a). We integrated XL-MS constraints^30^ with iterative AF3 modelling of candidate SKA3 segments, which converged on a high-confidence interface between the Ndc80C kink region and SKA3 residues 355–380 (Fig. 3b and Extended Data Fig. 12a,b). The predicted SKA3-Ndc80C contact interface closely matched the cryo-EM density, despite differences in the bend and rotational angle of the Ndc80C coiled-coil region itself (Extended Data Fig. 12c). This assignment is consistent with prior evidence that neighboring regions within the SKA3 C-terminal domain participate in MT-associated interactions^30,62^ and that residues 355–380 contribute to Ndc80C binding in solution^31^. Our structure visualized its engagement with the Ndc80C kink region in the MT-bound state (Fig. 3a–c). The bound SKA3 segment includes a short α-helix (“tether” helix) and two functionally important phosphorylation sites, T358 and T360 (corresponding to D358 and D360 in SkaC-6D; Fig. 3c and Supplementary Video 1). The interface is organized around an extended hydrophobic zipper between SKA3 and NDC80/NUF2 (Fig. 3b), with several of the contributing hydrophobic residues highly conserved in metazoans (Fig. 3e). T358 (D358 in our structure) is located by NDC80 H320 and NUF2 K213 (Fig. 3c), the latter corresponding to a hotspot identified by XL-MS^30^.

To validate this proposed interface, we generated a SkaC-6D mutant lacking this segment (SKA3 Δ355–380; SkaC-6D_DEL) and compared its MT-binding affinity with that of the full-length SkaC-6D (SkaC-6D_FL) in the presence or absence of Ndc80C (Fig. 3f–h). In MT pelleting assays, SkaC-6D_DEL and SkaC-6D_FL showed similar binding in the absence of Ndc80C (Fig. 3g), indicating that the deletion of this segment does not impair basal SkaC-MT association. By contrast, Ndc80C enhanced MT binding of SkaC-6D_FL, but reduced the binding of SkaC-6D_DEL, likely due to competition for an overlapping binding site on tubulin. Reciprocally, Ndc80C binding also diminished in the presence of SkaC-6D_DEL with respect to SkaC-6D_FL (Fig. 3h). These results indicate that the SKA3 355–380 segment is dispensable for basal SkaC-MT binding but is required for the cooperative binding of SkaC and Ndc80C on the MT surface. In the absence of this SkaC-Ndc80C coupling at the higher-radius coiled-coil region, the Ndc80C-CHD and SKA1-MTBD compete for MT binding, as indicated by the overlap on their tubulin footprint (Extended Data Fig. 5a,b and Supplementary Video 1). Together, these findings identify the SKA3 355–380 segment as a tether that links SkaC to the Ndc80C coiled-coil kink, thereby enabling cooperative binding of the two complexes on MTs.

### SKA3 tethering couples Ndc80C-SkaC to MT dynamics

**Fig. 4: |:**
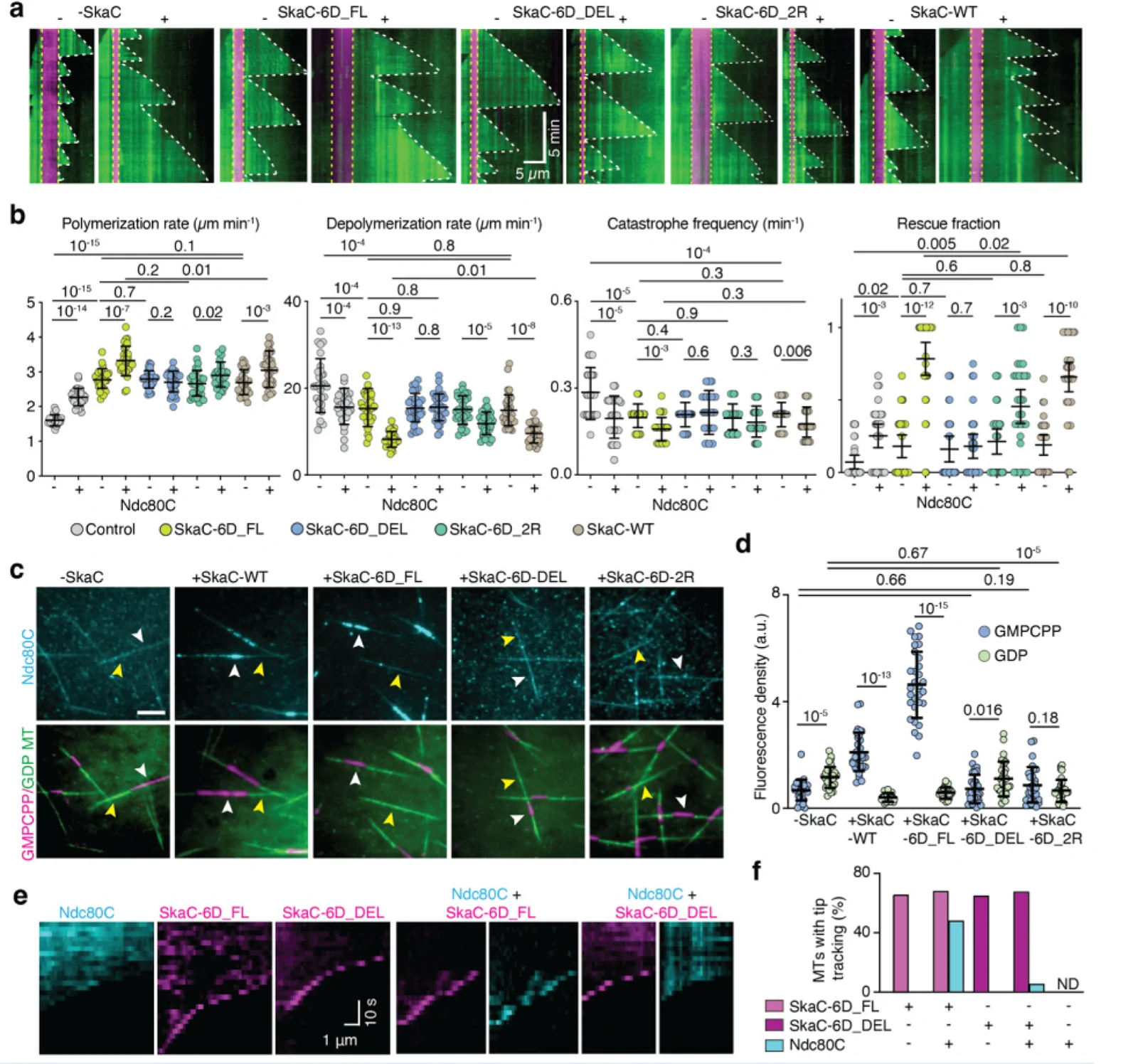
The SKA3 tether couples SkaC lattice selectivity to Ndc80C MT-end stabilization and tip tracking. **a,** Representative kymographs of dynamic MTs (green) in the presence of 50 nM Ndc80C-eGFP, 100 nM SkaC-6D or SkaC-6D mutants, 100 nM SkaC-WT alone or in combination, as indicated. Vertical, yellow dashed lines mark the ends of GMPCPP-stabilized seeds (magenta), and white traces mark the MT plus-ends. **b,** Quantification of MT plus end polymerization rate, depolymerization rate, catastrophe frequency, and rescue fraction under the indicated conditions. The center line and whiskers represent mean ± SD for polymerization rate, depolymerization rate, catastrophe frequency and mean with 95% CI of mean. P values were calculated using a two-tailed t-test. N = 30 MTs were analyzed for each condition from two independent experiments. **c,** Representative images showing binding of Ndc80C-eGFP (50 nM) in the presence of Ska-WT and SkaC-6D (or its mutants) on GMPCPP MT lattices (magenta) versus GDP lattices (green). White and yellow arrowheads indicate binding on the GMPCPP and GDP lattice, respectively. Images were individually contrast adjusted to visualize weak binding regions. Images were individually contrast adjusted to visualize weak binding regions. Scale bar: 5 μm. **d,** Integrated fluorescence density of Ndc80C under the indicated conditions. The center line and whiskers represent mean ± SD, respectively. P values were calculated using a two-tailed t-test. N = 30 MTs were analyzed for each condition from two independent experiments. **e,** Representative kymographs of depolymerizing MTs showing Ska6D_FL and Ska6D_DEL (magenta) in the presence or absence of Ndc80C (cyan), as well as Ndc80C alone (cyan). MT plus ends are oriented to the right. **f,** Quantification of the percentage of MTs exhibiting tip tracking by Ska6D_FL, Ska6D_DEL, and Ndc80C under the indicated conditions. ND: not determined. Data represent the mean from n = 35 MTs across N = 2 independent experiments.

We next examined how Ndc80C and SkaC affect MT dynamics (Fig. 4a,b). Compared to the control lacking either complex, both Ndc80C and SkaC alone measurably stabilized MTs by increasing polymerization rate, reducing depolymerization rate, decreasing catastrophe frequency, and increasing rescue frequency (Fig. 4a,b). SkaC had a stronger effect at the concentrations used in our assay. In the presence of both complexes, SkaC-WT enhanced Ndc80C-mediated stabilization of dynamic MT ends. This effect was further amplified in the presence of SkaC-6D_FL (Fig. 4a,b), consistent with its phosphorylation-enhanced interaction with Ndc80C^31,34,39^.

To determine how SKA3-Ndc80C interaction contributes to regulation of MT plus-end dynamics, we tested MT dynamics using a SKA3 deletion mutant lacking this tether segment (SkaC-6D_DEL. The absence of the tethering elements within SKA3 had no effect on the stabilization of the MTs for SkaC alone, but largely abolished the additional stabilizing effect observed together with Ndc80C (Fig. 4a,b). Mutation of the two phosphorylation sites within this region (R358/R360; SkaC-6D_2R) did not significantly alter the MT dynamics parameters observed for SkaC alone but exhibited an intermediate stabilizing effect relative to SkaC-6D_FL and SkaC-6D_DEL in the presence of Ndc80C (Fig. 4a,b). These results identify the SKA3 tether segment as contributing to the added effect of SkaC and Ndc80C in the reduction of dynamics at MT plus ends, with the two phosphorylation sites within this region fine-tuning this activity.

### The SKA3 tether couples SkaC lattice selectivity to Ndc80C tip tracking

We next asked whether the SKA3 tether also transmits SkaC-dependent lattice-state recognition to Ndc80C. In the presence of SkaC-6D_FL, Ndc80C showed a stronger preference for the GTP-like lattice than in the presence of SkaC-WT (Fig. 4c,d), consistent with phosphorylation-enhanced SkaC-Ndc80C coupling^31,34,39^. By contrast, the lattice bias was abolished in the presence of SkaC-6D_DEL and substantially reduced in SkaC-6D_2R, indicating that deletion or mutation of the tether disrupts the coupling required to bias Ndc80C binding. Thus, while the SKA1 MTBD provides lattice-state selectivity, the SKA3 tether is required to transmit this selectivity to Ndc80C.

Because persistent coupling to dynamic MT ends is a defining feature of KT function^5,24,29,63^, we next tested whether the same tether provides the physical link through which SkaC confers plus-end tracking on Ndc80C^29^. SkaC-dependent tracking of Ndc80C-associated assemblies had been observed previously^29,31^, but the structural basis for this activity remains unknown. In our MT depolymerization assay, SkaC robustly tracked depolymerizing MT tips, whereas Ndc80C alone showed no detectable tip-tracking activity under the same conditions (Fig. 4e,f). Full-length SkaC-6D (SkaC-6D_FL) recruited Ndc80C to depolymerizing MT ends and conferred tip-tracking behavior on Ndc80C. Deletion of the SKA3 tether (SkaC-6D_DEL) did not abolish SkaC tip tracking itself, but largely prevented SkaC from conferring this activity on Ndc80C. Together, these results identify the SKA3 tether as the structural conduit that transfers SkaC lattice selectivity and plus-end tracking to Ndc80C, converting Ndc80C into a Ska-coupled tip-tracking assembly.

## DISCUSSION

Our findings reveal how human SkaC couples Ndc80C to the structural state and dynamics of MT plus ends. In our structure, clustered Ndc80C on one PF positions SkaC on an adjacent PF through a phosphosite-containing SKA3 tether bound to a kink in the Ndc80C coiled coil. This arrangement brings the NDC80 CH domain and the SKA1 MTBD together around the α-CTT, forming a sandwich-like interface whose engagement is strengthened on polyglutamylated MTs. The SKA1 MTBD bridges the tubulin interdimer interface and preferentially recognizes the extended, GTP-like lattice characteristic of growing MT plus ends. Through the SKA3 tether, this SkaC-dependent lattice preference is transmitted to Ndc80C, promoting Ndc80C enrichment at the extended plus-end lattice, stabilization of dynamic MT ends, and Ndc80C tip tracking. Thus, the Ndc80C-SkaC interface provides a structural and regulatory mechanism for maintaining KT attachments that are strong enough to bear force yet adaptable enough to follow MT-end remodeling during chromosome segregation.

Our work identifies the tubulin tails as a tunable regulatory layer within the KT-MT attachment. Rather than serving only as flexible, negatively charged extensions of the MT surface, the α-tubulin C-terminal tail is structurally captured at the Ndc80C-SkaC interface, where its PTM state can tune assembly of the combined outer-KT MT-binding module. Removal of the tubulin tails weakens the binding of both Ndc80C and SkaC to MTs, and a stronger association to brain MTs versus HeLa MTs supports a positive contribution of tubulin PTMs to this interaction. Most specifically, the selective reduction of Ndc80C and SkaC recruitment on TTLL1-deficient, but not TTLL7-deficient MTs further argues that α-CTT polyglutamylation has a strongly positive effect in the binding of both Ndc80C and SkaC. These findings provide a molecular link between the tubulin code and outer-kinetochore attachment, suggesting that chromosome segregation can be regulated not only through phosphorylation of kinetochore proteins, but also through post-translational tuning of the microtubule lattice itself. Our data would also provide a mechanistic paradigm explaining previous findings demonstrating that loss of α-tubulin polyglutamylation catalyzed by TTLL11 leads to a high rate of chromosome segregation errors^44^, a common feature of kinetochore dysfunction^9,64^.

Our structures also clarify how human Ndc80C forms a cooperative MT-bound scaffold for SkaC engagement. Multivalent Ndc80C assemblies are required for efficient tip tracking and force coupling^63^, but the molecular basis for human Ndc80C cooperativity has remained incompletely defined. The NDC80 CHD engages the NDC80 NTT and NUF2 of a neighboring Ndc80C, templated by the MT lattice. Based on our results and previous studies^12,14,59,63^, we propose that the human Ndc80C cooperativity is nucleated via NDC80 CHD- and NTT-mediated interactions near the MT surface, and then propagates along the coiled-coil, generating a robust arrangement with the multivalency needed for sustained MT engagement and load bearing. This organization provides a metazoan solution to robust KT-MT coupling. Whereas budding-yeast Ndc80C gains multivalency through association with the oligomeric Dam1C ring^20^, human Ndc80C forms an intrinsic cooperative scaffold that positions dimeric SkaC on adjacent PFs. Thus, distinct eukaryotic lineages appear to have evolved different molecular architectures to meet the shared challenge of maintaining attachment to dynamic MT ends^1,2^.

**Fig. 5: |:**
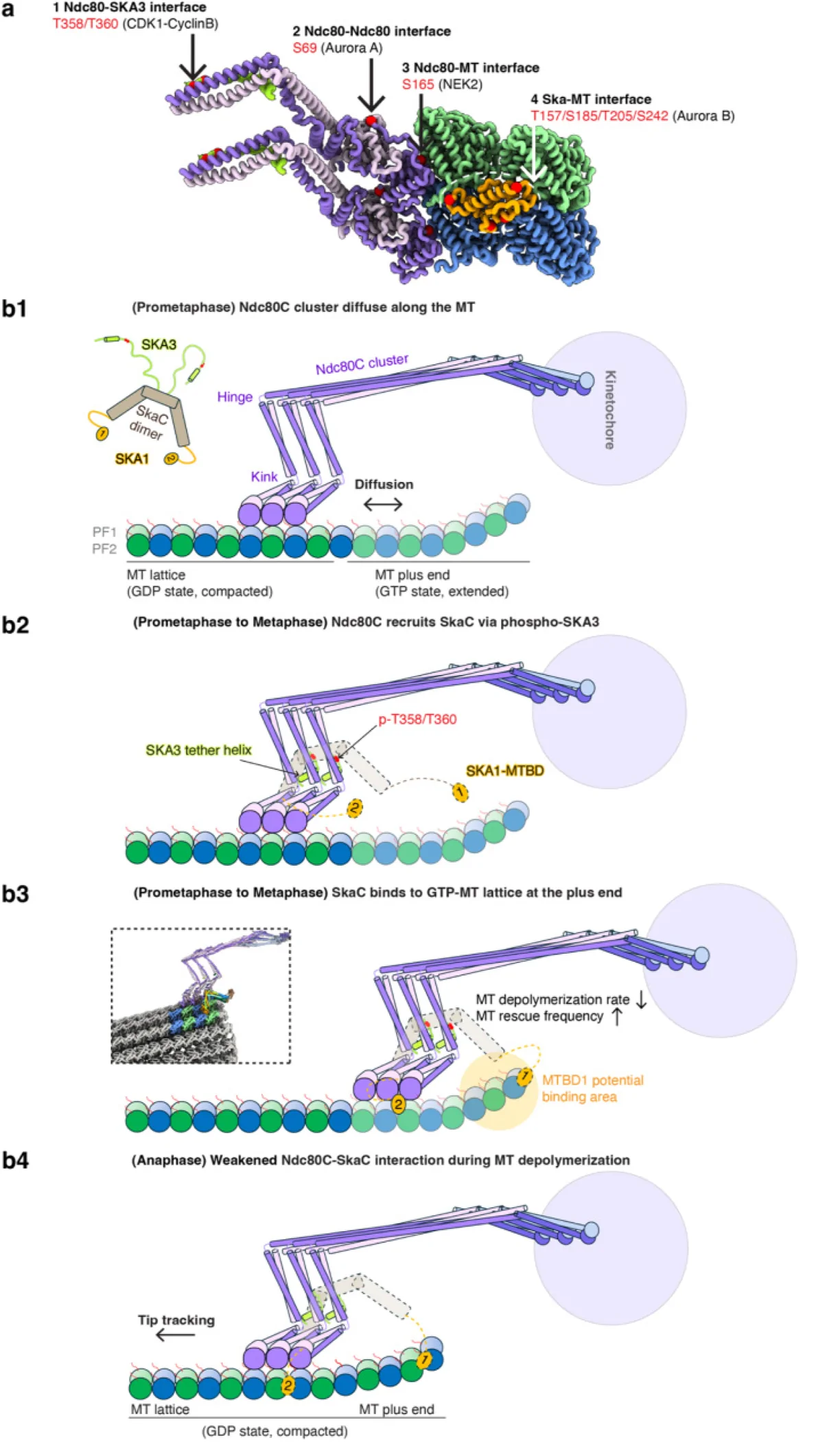
Regulatory phosphosites and a mechanistic model of Ndc80C-SkaC coordination at MT plus ends during mitosis. **a,** Structurally resolved regulatory phosphorylation sites (red spheres) relative to the MT-binding surfaces, adjacent Ndc80C interfaces, and the SKA3-Ndc80C interface (see also Supplementary Video 1). **b,** In prometaphase and metaphase, clustered Ndc80C recruits SkaC to adjacent PFs through the binding of the SKA3 tether segment to the Ndc80C kink region (**b1-b2**). SKA1 MTBD binds next to Ndc80C, stabilizes the MT end, and biases the assembly toward the extended GTP-like lattice at MT plus ends (**b3**). This coupling is expected to weaken in anaphase, as MT ends shorten and mitotic phosphorylation declines (**b4**). (α-tubulin (green), β-tubulin (blue), NDC80 (medium purple), NUF2 (thistle), SPC24 (slate blue), SPC25 (light blue)). SKA1 MTBDs are colored orange, and SKA3 C-terminal tails are colored green-yellow; SKA1–2-3 coiled-coil bundles are simplified as tan rectangles, and their positional uncertainty is indicated with dashed lines and reduced opacity. The dashed rectangle in B3 shows a more detailed model of the state described in our study.

Our structural findings provide a framework for understanding how mitotic phosphorylation regulates this attachment interface. Several regulatory phosphosites map directly to MT-binding, Ndc80C-Ndc80C, or Ndc80C-SkaC interfaces (Fig. 5a and Supplementary Video 1). NDC80 S165 defines a NEK2-regulated node at the CHD-MT interface, in line with its reported roles in chromosome alignment and spindle-checkpoint signaling^52–54^. Aurora B-sensitive sites in SKA1 within or immediately adjacent to its MT binding interface are consistent with Aurora B function in earlymitotic error correction and in restraining premature SkaC accumulation at KTs^27,29,33,65^. NDC80 S69 lies at the interface with a neighboring Ndc80C, providing a structural rationale for how Aurora A could dampen Ndc80 clustering to regulate metaphase KT-MT dynamics^55^, and consistent with MT-bound Ndc80 oligomerization to modulate Aurora kinase access to Ndc80C phosphosites^66^. Finally, SKA3 T358 and T360 map directly to the SKA3-Ndc80C kink interface, consistent with CDK1-dependent phosphorylation promoting SKA3 binding to Ndc80C and SkaC recruitment to KTs^31,34^. These observations suggest that mitotic kinases regulate KT-MT attachment not through a single switch, but by tuning multiple contact points within the Ndc80C-SkaC-MT assembly.

The functional significance of this architecture is most apparent at dynamic MT plus ends, where KTs must remain attached while the underlying tubulin lattice continuously remodels. SkaC preferentially associates with the extended, GTP-like lattice by engaging the tubulin interdimer interface, whereas Ndc80C alone remains more broadly distributed on MTs. Through the SKA3 tether, this lattice preference is transmitted to Ndc80C, providing a mechanism for enriching the core force-coupling complex at the MT-end for persistent attachment. This interpretation helps explain previous observations that Ndc80C-bound SkaC stabilizes MT ends in a stalled state^31^, that SkaC can autonomously track growing plus ends^42^, and that a tripartite Ndc80-Cdt1-Ska complex supports processive bidirectional plus-end tracking^67^. Importantly, our tip-tracking assays revealed that the SKA3 tether provides a physical link that allows SkaC to transfer MT-end recognition to Ndc80C, enabling regulated attachment that can remain coupled to dynamic MT plus ends at KTs. Recent cellular work showed that perturbing an overlapping SKA3 C-terminal segment delays mitotic progression, supporting the physiological relevance of the SKA3 tether region structurally defined here^68^.

Previous cellular studies established that Ndc80C is recruited to KT early in mitosis^69,70^ before stable end-on attachments form^4^. In comparison, SkaC becomes strongly enriched following MT attachment^23,25,27^, increases progressively as KTs congress and biorient^26^, and reaches maximal levels at metaphase^27,71^. Our structural and functional data support a stepwise model for how coordinated Ndc80C-SkaC interactions maintain KT attachment to dynamic MT ends while modulating MT behavior (Fig. 5b). In prometaphase, KT-bound Ndc80C captures spindle MTs, forms cooperative clusters along PFs, and diffuses on the lattice (Fig. 5b1). As cells progress through prometaphase and metaphase, when CDK1 activity is high^72^, phosphorylated SkaC is recruited through the SKA3-Ndc80C kink interface (Fig. 5b2). In this arrangement, one SKA1 MTBD lies adjacent to Ndc80C and, together with the NDC80 CHD, stabilizes the α-CTT “sandwich” interface, and biases Ndc80C towards the extended GTP-like lattice at the MT plus end (Fig. 5b3). A second SKA1 MTBD may engage with the curved end-associated tubulin states, as previously proposed^33,42^, using a distinct surface from that used to interact with the straight MT lattice (Extended Data Fig. 11g–i). Through the SKA3 tether, this SkaC-dependent lattice preference is transmitted to Ndc80C, enabling Ndc80C tip tracking and stabilizing dynamic MT ends. This Ndc80C-SkaC arrangement is not necessarily a long-lived static assembly but can be dynamically renewed to maintain a coupling state at the MT interface^70,73^. As cells enter anaphase, depolymerizing MT ends and declining CDK1 activity^74^ may jointly disfavor SKA1 engagement with MTs and weaken phospho-dependent SKA3-Ndc80C interaction (Fig. 5b4), in line with the reduced KT abundance of SKA3 at this stage^75^. Together, this model integrates cooperative clustering, phospho-regulated recruitment, and lattice-state recognition for metazoan KTs to maintain persistent yet adaptable coupling to dynamic MT ends.

Our findings define the outer KT as a coordinated, multivalent molecular assembly that actively “reads” the lattice state to navigate the dynamic mitotic environment. Ultimately, this phospho-regulated and geometry-dependent coupling ensures the high-fidelity chromosome segregation necessary for organismal viability, while its disruption provides a mechanistic path toward the aneuploidy and chromosome instability observed in human disease^9,10,76^.

## Supplementary Material

Supplement 1

## Data Availability

The composite Tubulin-Ndc80-Ska map, the Tubulin-Ndc80-Ska consensus map, and focused maps of β-tubulin, two adjacent Ndc80 complexes, and the Ndc80 and SKA3 have been deposited in the Electron Microscopy Data Bank (EMDB) under accession codes 75942, 75938, 75939, 75940 and 75941, respectively. The coordinates for the corresponding models have been deposited in the Protein Data Bank (PDB) with accession code 11QA.

## References

[1] Cheeseman I.M. & Desai A. Molecular architecture of the kinetochore–microtubule interface. Nature Reviews Molecular Cell Biology 9, 33–46 (2008). 10.1038/nrm2310 18097444

[2] Musacchio A. & Desai A. A Molecular View of Kinetochore Assembly and Function. Biology (Basel) 6(2017). 10.3390/biology6010005 PMC5371998 28125021

[3] Bakhoum S.F., Thompson S.L., Manning A.L. & Compton D.A. Genome stability is ensured by temporal control of kinetochore-microtubule dynamics. Nat Cell Biol 11, 27–35 (2009). 10.1038/ncb1809 PMC2614462 19060894

[4] DeLuca J.G. Kinetochore Microtubule Dynamics and Attachment Stability Are Regulated by Hec1. Cell 127, 969–982 (2006). 10.1016/j.cell.2006.09.047 17129782

[5] Powers A.F. The Ndc80 Kinetochore Complex Forms Load-Bearing Attachments to Dynamic Microtubule Tips via BiasedDiffusion. Cell 136, 865–875 (2009). 10.1016/j.cell.2008.12.045 PMC2749323 19269365

[6] Akiyoshi B. Tension directly stabilizes reconstituted kinetochore-microtubule attachments. Nature 468, 576–9 (2010). 10.1038/nature09594 PMC3108429 21107429

[7] Cimini D. Merotelic kinetochore orientation is a major mechanism of aneuploidy in mitotic mammalian tissue cells. J Cell Biol 153, 517–27 (2001). 10.1083/jcb.153.3.517 PMC2190575 11331303

[8] Cimini D. Merotelic kinetochore orientation, aneuploidy, and cancer. Biochimica et Biophysica Acta (BBA) - Reviews on Cancer 1786, 32–40 (2008). 10.1016/j.bbcan.2008.05.003 18549824

[9] Bakhoum S.F., Genovese G. & Compton D.A. Deviant kinetochore microtubule dynamics underlie chromosomal instability. Curr Biol 19, 1937–42 (2009). 10.1016/j.cub.2009.09.055 PMC2787757 19879145

[10] Santaguida S. & Amon A. Short- and long-term effects of chromosome mis-segregation and aneuploidy. Nature Reviews Molecular Cell Biology 16, 473–485 (2015). 10.1038/nrm4025 26204159

[11] Cheeseman I.M., Chappie J.S., Wilson-Kubalek E.M. & Desai A. The conserved KMN network constitutes the core microtubule-binding site of the kinetochore. Cell 127, 983–97 (2006). 10.1016/j.cell.2006.09.039 17129783

[12] Ciferri C. Implications for Kinetochore-Microtubule Attachment from the Structure of an Engineered Ndc80 Complex. Cell 133, 427–439 (2008). 10.1016/j.cell.2008.03.020 PMC4754795 18455984

[13] Ciferri C. Architecture of the Human Ndc80-Hec1 Complex, a Critical Constituent of the Outer Kinetochore*. Journal of Biological Chemistry 280, 29088–29095 (2005). 10.1074/jbc.M504070200 15961401

[14] Alushin G.M. The Ndc80 kinetochore complex forms oligomeric arrays along microtubules. Nature 467, 805–810 (2010). 10.1038/nature09423 PMC2957311 20944740

[15] Petrovic A. The MIS12 complex is a protein interaction hub for outer kinetochore assembly. J Cell Biol 190, 835–52 (2010). 10.1083/jcb.201002070 PMC2935574 20819937

[16] Huis in ’t Veld P.J., Molecular basis of outer kinetochore assembly on CENP-T. eLife 5, e21007 (2016). 10.7554/eLife.21007 PMC5241120 28012276

[17] Westermann S. Formation of a dynamic kinetochore- microtubule interface through assembly of the Dam1 ring complex. Mol Cell 17, 277–90 (2005). 10.1016/j.molcel.2004.12.019 15664196

[18] Wang H.-W. Architecture of the Dam1 kinetochore ring complex and implications for microtubule-driven assembly and force-coupling mechanisms. Nature Structural & Molecular Biology 14, 721–726 (2007). 10.1038/nsmb1274 17643123

[19] Jenni S. & Harrison S.C. Structure of the DASH/Dam1 complex shows its role at the yeast kinetochore-microtubule interface. Science 360, 552–558 (2018). 10.1126/science.aar6436 PMC6815591 29724956

[20] Muir K.W. Structural mechanism of outer kinetochore Dam1-Ndc80 complex assembly on microtubules. Science 382, 1184–1190 (2023). 10.1126/science.adj8736 PMC7615550 38060647

[21] Janke C. The budding yeast proteins Spc24p and Spc25p interact with Ndc80p and Nuf2p at the kinetochore and are important for kinetochore clustering and checkpoint control. Embo j 20, 777–91 (2001). 10.1093/emboj/20.4.777 PMC145434 11179222

[22] Janke C., Ortíz J., Tanaka T.U., Lechner J. & Schiebel E. Four new subunits of the Dam1-Duo1 complex reveal novel functions in sister kinetochore biorientation. Embo j 21, 181–93 (2002). 10.1093/emboj/21.1.181 PMC125813 11782438

[23] Hanisch A., Silljé H.H.W. & Nigg E.A. Timely anaphase onset requires a novel spindle and kinetochore complex comprising Ska1 and Ska2. The EMBO Journal 25, 5504–5515-5515 (2006). 10.1038/sj.emboj.7601426 PMC1679759 17093495

[24] Welburn J.P. The human kinetochore Ska1 complex facilitates microtubule depolymerization-coupled motility. Dev Cell 16, 374–85 (2009). 10.1016/j.devcel.2009.01.011 PMC2746561 19289083

[25] Gaitanos T.N. Stable kinetochore–microtubule interactions depend on the Ska complex and its new component Ska3/C13Orf3. The EMBO Journal 28, 1442–1452-1452 (2009). 10.1038/emboj.2009.96 PMC2669960 19360002

[26] Auckland P., Clarke N.I., Royle S.J. & McAinsh A.D. Congressing kinetochores progressively load Ska complexes to prevent force-dependent detachment. Journal of Cell Biology 216, 1623–1639 (2017). 10.1083/jcb.201607096 PMC5461014 28495837

[27] Chan Y.W., Jeyaprakash A.A., Nigg E.A. & Santamaria A. Aurora B controls kinetochore-microtubule attachments by inhibiting Ska complex-KMN network interaction. J Cell Biol 196, 563–71 (2012). 10.1083/jcb.201109001 PMC3307695 22371557

[28] Zhang G. The Ndc80 internal loop is required for recruitment of the Ska complex to establish end-on microtubule attachment to kinetochores. Journal of Cell Science 125, 3243–3253 (2012). 10.1242/jcs.104208 22454517

[29] Schmidt Jens C. The Kinetochore-Bound Ska1 Complex Tracks Depolymerizing Microtubules and Binds to Curved Protofilaments. Developmental Cell 23, 968–980 (2012). 10.1016/j.devcel.2012.09.012 PMC3500403 23085020

[30] Helgeson L.A. Human Ska complex and Ndc80 complex interact to form a load-bearing assembly that strengthens kinetochore–microtubule attachments. Proceedings of the National Academy of Sciences 115, 2740–2745 (2018). 10.1073/pnas.1718553115 PMC5856539 29487209

[31] Huis In ’t Veld P.J., Volkov V.A., Stender I.D., Musacchio A. & Dogterom M. Molecular determinants of the Ska-Ndc80 interaction and their influence on microtubule tracking and force-coupling. Elife 8(2019). 10.7554/eLife.49539 PMC6927755 31804178

[32] Jeyaprakash A.A. Structural and Functional Organization of the Ska Complex, a Key Component of the Kinetochore-Microtubule Interface. Molecular Cell 46, 274–286 (2012). 10.1016/j.molcel.2012.03.005 22483620

[33] Abad M.A. Structural basis for microtubule recognition by the human kinetochore Ska complex. Nature Communications 5, 2964 (2014). 10.1038/ncomms3964 PMC3923297 24413531

[34] Zhang Q. Ska3 Phosphorylated by Cdk1 Binds Ndc80 and Recruits Ska to Kinetochores to Promote Mitotic Progression. Current Biology 27, 1477–1484.e4 (2017). 10.1016/j.cub.2017.03.060 28479321

[35] Janczyk P. Mechanism of Ska Recruitment by Ndc80 Complexes to Kinetochores. Dev Cell 41, 438–449.e4 (2017). 10.1016/j.devcel.2017.04.020 PMC5926205 28535377

[36] Cheerambathur D.K. Dephosphorylation of the Ndc80 Tail Stabilizes Kinetochore-Microtubule Attachments via the SkaComplex. Developmental Cell 41, 424–437.e4 (2017). 10.1016/j.devcel.2017.04.013 PMC5572820 28535376

[37] Wimbish R.T. The Hec1/Ndc80 tail domain is required for force generation at kinetochores, but is dispensable for kinetochore-microtubule attachment formation and Ska complex recruitment. Mol Biol Cell 31, 1453–1473 (2020). 10.1091/mbc.E20-05-0286 PMC7359571 32401635

[38] Radhakrishnan R.M., Stokes L., Day M., Huis in ’t Veld P.J. & Volkov V.A. Microtubule end stabilisation by cooperative oligomers of Ska and Ndc80 complexes. The EMBO Journal (2026). 10.1038/s44318-026-00749-5 PMC13144512 41862644

[39] Zhang Q., Hu L., Chen Y., Tian W. & Liu H. Multisite phosphorylation determines the formation of Ska-Ndc80 macro-complexes that are essential for chromosome segregation during mitosis. Mol Biol Cell 31, 1892–1903 (2020). 10.1091/mbc.E19-10-0569 PMC7525821 32491969

[40] Alushin G.M. Multimodal microtubule binding by the Ndc80 kinetochore complex. Nat Struct Mol Biol 19, 1161–7 (2012). 10.1038/nsmb.2411 PMC3492541 23085714

[41] Tooley J.G., Miller S.A. & Stukenberg P.T. The Ndc80 complex uses a tripartite attachment point to couple microtubule depolymerization to chromosome movement. Mol Biol Cell 22, 1217–26 (2011). 10.1091/mbc.E10-07-0626 PMC3078066 21325630

[42] Monda J.K. Microtubule Tip Tracking by the Spindle and Kinetochore Protein Ska1 Requires Diverse Tubulin-Interacting Surfaces. Curr Biol 27, 3666–3675.e6 (2017). 10.1016/j.cub.2017.10.018 PMC5726585 29153323

[43] Welburn J.P.I. The Human Kinetochore Ska1 Complex Facilitates Microtubule Depolymerization-Coupled Motility. Developmental Cell 16, 374–385 (2009). 10.1016/j.devcel.2009.01.011 PMC2746561 19289083

[44] Zadra I. Chromosome segregation fidelity requires microtubule polyglutamylation by the cancer downregulated enzyme TTLL11. Nature Communications 13, 7147 (2022). 10.1038/s41467-022-34909-y PMC9681853 36414642

[45] Ruse C., Chin H. & Pradhan S. Polyglutamylation: biology and analysis. Amino Acids 54(2022). 10.1007/s00726-022-03146-4 PMC9117372 35357568

[46] Natarajan K., Gadadhar S., Souphron J., Magiera M.M. & Janke C. Molecular interactions between tubulin tails and glutamylases reveal determinants of glutamylation patterns. EMBO Rep 18, 1013–1026 (2017). 10.15252/embr.201643751 PMC5452014 28483842

[47] Eddé B. Posttranslational glutamylation of alpha-tubulin. Science 247, 83–5 (1990). 10.1126/science.1967194 1967194

[48] Souphron J. Purification of tubulin with controlled post-translational modifications by polymerization-depolymerization cycles. Nat Protoc 14, 1634–1660 (2019). 10.1038/s41596-019-0153-7 30996262

[49] Janke C. The tubulin code: molecular components, readout mechanisms, and functions. J Cell Biol 206, 461–72 (2014). 10.1083/jcb.201406055 PMC4137062 25135932

[50] Bodakuntla S. Distinct roles of α- and β-tubulin polyglutamylation in controlling axonal transport and in neurodegeneration. Embo j 40, e108498 (2021). 10.15252/embj.2021108498 PMC8408597 34309047

[51] Ping Y. Tubulin Polyglutamylation by TTLL1 and TTLL7 Regulate Glutamate Concentration in the Mice Brain. Biomolecules 13, 784 (2023). 10.3390/biom13050784 PMC10216738 37238654

[52] Chen Y., Riley D.J., Zheng L., Chen P.-L. & Lee W.-H. Phosphorylation of the Mitotic Regulator Protein Hec1 by Nek2 Kinase Is Essential for Faithful Chromosome Segregation*. Journal of Biological Chemistry 277, 49408–49416 (2002). 10.1074/jbc.M207069200 12386167

[53] Du J. The mitotic checkpoint kinase NEK2A regulates kinetochore microtubule attachment stability. Oncogene 27, 4107– 4114 (2008). 10.1038/onc.2008.34 PMC7983366 18297113

[54] Wei R., Ngo B., Wu G. & Lee W.H. Phosphorylation of the Ndc80 complex protein, HEC1, by Nek2 kinase modulates chromosome alignment and signaling of the spindle assembly checkpoint. Mol Biol Cell 22, 3584–94 (2011). 10.1091/mbc.E11-01-0012 PMC3183014 21832156

[55] DeLuca K.F. Aurora A kinase phosphorylates Hec1 to regulate metaphase kinetochore–microtubule dynamics. Journal of Cell Biology 217, 163–177 (2017). 10.1083/jcb.201707160 PMC5748988 29187526

[56] Wang H.-W. Architecture and Flexibility of the Yeast Ndc80 Kinetochore Complex. Journal of Molecular Biology 383, 894–903 (2008). 10.1016/j.jmb.2008.08.077 PMC2640231 18793650

[57] Scarborough E.A., Davis T.N. & Asbury C.L. Tight bending of the Ndc80 complex provides intrinsic regulation of its binding tomicrotubules. Elife 8(2019). 10.7554/eLife.44489 PMC6516834 31045495

[58] Zahm J.A., Jenni S. & Harrison S.C. Structure of the Ndc80 complex and its interactions at the yeast kinetochore–microtubule interface. Open Biology 13, 220378 (2023). 10.1098/rsob.220378 PMC9993044 36883282

[59] Polley S. Stable kinetochore-microtubule attachment requires loop-dependent Ndc80-Ndc80 binding. Embo j 42, e112504 (2023). 10.15252/embj.2022112504 PMC10308368 37203876

[60] Abramson J. Accurate structure prediction of biomolecular interactions with AlphaFold 3. Nature 630, 493–500 (2024). 10.1038/s41586-024-07487-w PMC11168924 38718835

[61] Alushin Gregory M. High-Resolution Microtubule Structures Reveal the Structural Transitions in αβ-Tubulin upon GTP Hydrolysis. Cell 157, 1117–1129 (2014). 10.1016/j.cell.2014.03.053 PMC4054694 24855948

[62] Abad M.A. Ska3 Ensures Timely Mitotic Progression by Interacting Directly With Microtubules and Ska1 Microtubule Binding Domain. Scientific Reports 6, 34042 (2016). 10.1038/srep34042 PMC5036024 27667719

[63] Volkov V.A., Huis in ’t Veld, P.J., Dogterom, M. & Musacchio, A. Multivalency of NDC80 in the outer kinetochore is essential to track shortening microtubules and generate forces. eLife 7, e36764 (2018). 10.7554/eLife.36764 PMC5940359 29629870

[64] Thompson S.L. & Compton D.A. Chromosome missegregation in human cells arises through specific types of kinetochore-microtubule attachment errors. Proc Natl Acad Sci U S A 108, 17974–8 (2011). 10.1073/pnas.1109720108 PMC3207692 21997207

[65] Hauf S. The small molecule Hesperadin reveals a role for Aurora B in correcting kinetochore-microtubule attachment and inmaintaining the spindle assembly checkpoint. J Cell Biol 161, 281–94 (2003). 10.1083/jcb.200208092 PMC2172906 12707311

[66] Niu Y. Microtubules guide Aurora B substrate geometries for accurate chromosome segregation. Science Advances 12, eaea2112 (2026). 10.1126/sciadv.aea2112 PMC13015866 41880487

[67] Rahi A. The Ndc80-Cdt1-Ska1 complex is a central processive kinetochore-microtubule coupling unit. J Cell Biol 222(2023). 10.1083/jcb.202208018 PMC10238862 37265445

[68] Daum J.R., Romek N. & Gorbsky G.J. Functional Interaction of SKA and NDC80 Complexes at Kinetochores Promoting Anaphase Onset in Mitosis. bioRxiv, 2026.05.22.727258 (2026). 10.1091/mbc.E26-05-0235 42987094

[69] DeLuca J.G. Hec1 and nuf2 are core components of the kinetochore outer plate essential for organizing microtubule attachment sites. Mol Biol Cell 16, 519–31 (2005). 10.1091/mbc.E04-09-0852 PMC545888 15548592

[70] Hori T., Haraguchi T., Hiraoka Y., Kimura H. & Fukagawa T. Dynamic behavior of Nuf2-Hec1 complex that localizes to the centrosome and centromere and is essential for mitotic progression in vertebrate cells. J Cell Sci 116, 3347–62 (2003). 10.1242/jcs.00645 12829748

[71] Sivakumar S. & Gorbsky G.J. Phosphatase-regulated recruitment of the spindle- and kinetochore-associated (Ska) complex to kinetochores. Biol Open 6, 1672–1679 (2017). 10.1242/bio.026930 PMC5703607 28982702

[72] Gavet O. & Pines J. Progressive Activation of CyclinB1-Cdk1 Coordinates Entry to Mitosis. Developmental Cell 18, 533–543 (2010). 10.1016/j.devcel.2010.02.013 PMC3325599 20412769

[73] Raaijmakers J.A., Tanenbaum M.E., Maia A.F. & Medema R.H. RAMA1 is a novel kinetochore protein involved in kinetochore-microtubule attachment. Journal of Cell Science 122, 2436–2445 (2009). 10.1242/jcs.051912 19549680

[74] Afonso O. Spatiotemporal control of mitotic exit during anaphase by an aurora B-Cdk1 crosstalk. Elife 8(2019). 10.7554/eLife.47646 PMC6706241 31424385

[75] Chen Y.-C., Kilic E., Wang E., Rossman W. & Suzuki A. CENcyclopedia: dynamic landscape of kinetochore architecture throughout the cell cycle. Nature Communications 16, 7676 (2025). 10.1038/s41467-025-62316-6 PMC12361584 40825761

[76] Uiterkamp M.S. The SKA complex is a mitotic vulnerability of chromosomally unstable cancers. bioRxiv, 2026.05.21.726809 (2026).

[77] Mastronarde D.N. Automated electron microscope tomography using robust prediction of specimen movements. J Struct Biol 152, 36–51 (2005). 10.1016/j.jsb.2005.07.007 16182563

[78] Zivanov J. New tools for automated high-resolution cryo-EM structure determination in RELION-3. eLife 7, e42166 (2018). 10.7554/eLife.42166 PMC6250425 30412051

[79] Scheres S.H.W. RELION: Implementation of a Bayesian approach to cryo-EM structure determination. Journal of Structural Biology 180, 519–530 (2012). 10.1016/j.jsb.2012.09.006 PMC3690530 23000701

[80] Rohou A. & Grigorieff N. CTFFIND4: Fast and accurate defocus estimation from electron micrographs. J Struct Biol 192, 216–21 (2015). 10.1016/j.jsb.2015.08.008 PMC6760662 26278980

[81] Ferro L.S. Structural and functional insight into regulation of kinesin-1 by microtubule-associated protein MAP7. Science 375, 326–331 (2022). 10.1126/science.abf6154 PMC8985661 35050657

[82] Cook A.D., Manka S.W., Wang S., Moores C.A. & Atherton J. A microtubule RELION-based pipeline for cryo-EM image processing. Journal of Structural Biology 209, 107402 (2020). 10.1016/j.jsb.2019.10.004 PMC6961209 31610239

[83] Grigorieff N. Chapter Eight - Frealign: An Exploratory Tool for Single-Particle Cryo-EM. in Methods in Enzymology, Vol. 579 (ed. Crowther R.A.) 191–226 (Academic Press, 2016). 10.1016/bs.mie.2016.04.013 PMC6760665 27572728

[84] Zhang R. & Nogales E. A new protocol to accurately determine microtubule lattice seam location. Journal of Structural Biology 192, 245–254 (2015). 10.1016/j.jsb.2015.09.015 PMC4634897 26424086

[85] Wang N. Architecture of African swine fever virus and implications for viral assembly. Science 366, 640–644 (2019). 10.1126/science.aaz1439 31624094

[86] Sanchez-Garcia R. DeepEMhancer: a deep learning solution for cryo-EM volume post-processing. Communications Biology 4, 874 (2021). 10.1038/s42003-021-02399-1 PMC8282847 34267316

[87] Zhang R., LaFrance B. & Nogales E. Separating the effects of nucleotide and EB binding on microtubule structure. Proceedings of the National Academy of Sciences 115, E6191–E6200 (2018). 10.1073/pnas.1802637115 PMC6142192 29915050

[88] Goddard T.D. UCSF ChimeraX: Meeting modern challenges in visualization and analysis. Protein Sci 27, 14–25 (2018). 10.1002/pro.3235 PMC5734306 28710774

[89] Meng E.C. UCSF ChimeraX: Tools for structure building and analysis. Protein Science 32, e4792 (2023). 10.1002/pro.4792 PMC10588335 37774136

[90] Emsley P., Lohkamp B., Scott W.G. & Cowtan K. Features and development of Coot. Acta Crystallogr D Biol Crystallogr 66, 486–501 (2010). 10.1107/S0907444910007493 PMC2852313 20383002

[91] Croll T.I. ISOLDE: a physically realistic environment for model building into low-resolution electron-density maps. Acta Crystallogr D Struct Biol 74, 519–530 (2018). 10.1107/S2059798318002425 PMC6096486 29872003

[92] Liebschner D. Macromolecular structure determination using X-rays, neutrons and electrons: recent developments in Phenix. Acta Crystallogr D Struct Biol 75, 861–877 (2019). 10.1107/S2059798319011471 PMC6778852 31588918

[93] Larkin M.A. Clustal W and Clustal X version 2.0. Bioinformatics 23, 2947–8 (2007). 10.1093/bioinformatics/btm404 17846036

[94] Robert X. & Gouet P. Deciphering key features in protein structures with the new ENDscript server. Nucleic Acids Research 42, W320–W324 (2014). 10.1093/nar/gku316 PMC4086106 24753421

