# Supplementary material for: Structural Basis of Human Kinetochore-Microtubule Coupling by the Ndc80 and Ska Complexes
Source: bioRxiv. 2026 Jun 25:2025.12.06.692780. Originally published 2025 Dec 7. Preprint. [Version 2] doi: 10.64898/2025.12.06.692780 (PMC12822813; doi:10.64898/2025.12.06.692780)
Supplement: Supplementary file 1 [file media-1.docx]

**Methods**

**Cloning, expression, and purification of SkaC and Ndc80C**

All SkaC and Ndc80C genes were cloned into the pETDuet vector, with or without an N-terminal 14×His-BD Sumo tag. SkaC mutants were generated via PCR-based site-directed mutagenesis or gene synthesis. All sequences were verified by whole plasmid sequencing prior to expression. Constructs were expressed in *E. coli* BL21(DE3) RIL or Rosetta2 (DE3) pLysS strains grown in Luria Broth supplemented with kanamycin, ampicillin, and chloramphenicol. Protein expression was induced with 0.4 mM isopropyl β-D-1-thiogalactopyranoside (IPTG) at 18 °C for 18-20 hours. Cells were harvested by centrifugation and resuspended in lysis buffer (25 mM Tris-HCl pH 8.0, 400 mM NaCl, 20 mM imidazole, 5% glycerol, 5 mM 2-mercaptoethanol) supplemented with protease inhibitor cocktail tablets and 1 mM PMSF. Cells were lysed by sonication and lysates were clarified by centrifugation at 40,000 rpm in a Ti45 rotor (Beckman) for 1 hour. The supernatant was incubated with Ni-NTA resin (GoldBio) for 2 hours, followed by washing with wash buffer (25 mM Tris-HCl pH 8.0, 150 mM NaCl, 40 mM imidazole, 5% glycerol, 5 mM 2-mercaptoethanol). The His tag was cleaved on-column overnight using Sumo protease. Proteins were further purified by ion-exchange chromatography using a HiTrap Q column (Cytiva), followed by size-exclusion chromatography on a Superdex 200 Increase 10/300 column (Cytiva) equilibrated with gel filtration buffer (25 mM Tris-HCl, pH 8.0, 150 mM NaCl, 5% glycerol, 1 mM DTT). Peak fractions were concentrated, flash frozen in liquid nitrogen, and stored at -80°C. The procedures above were used for all SkaC and Ndc80C purification.

**Tubulin purification**

Tubulin was purified from wild-type and knock-out mouse brains or HeLa S3 cells grown in suspension using the polymerization/depolymerization protocol described previously^48^. In short, brains from wild-type (WT), Ttll1^-/-^, Ttll7^-/-^ or Ttll1^-/-^Ttll7^-/-^ mice were homogenized in lysis buffer (80 mM PIPES pH 6.9, 1 mM EGTA, 1 mM MgCl_2_, 1 mM 2-mercaptoethanol, 1 mM PMSF, and protease inhibitors), or HeLa cells were diluted in ice-cold lysis buffer and lysed using a French press. After homogenization, the lysates were ultracentrifuged (180,000 x g for brain tubulin or 112,000 x g for HeLa tubulin at 4 °C for 30 min), the supernatant was used for the first polymerization round in 1 mM GTP, 30% glycerol at 30 °C for 30 min. Polymerized MTs were pelleted at 180,000 x g, 30 °C for 30 min and subsequently depolymerized on ice for 20 min. The second polymerization/depolymerization cycle was performed in 0.5 M PIPES-KOH pH 6.8 buffer and the third cycle was performed as the first cycle. Purified tubulin was snap frozen and kept at -80 °C until MT assembly.

**MT polymerization**

MT preparation for EM sample: Porcine brain tubulin (Cytoskeleton) was reconstituted to 10 mg/ml in BRB80 buffer (80 mM PIPES, pH 6.9, 1 mM ethylene glycol tetraacetic acid (EGTA), pH 8.0, 1 mM MgCl₂) supplemented with 10% (v/v) glycerol, 1 mM GTP, and 1 mM DTT. Tubulin polymerization was initiated by incubating 10 µL of the solution at 37 °C for 30 minutes. MTs were pelleted by centrifugation at 20,000 × *g* for 20 minutes. The supernatant was carefully removed, and the pellet was resuspended in 8 µL of cold BRB80 buffer supplemented with 1.25 mM GMPCPP (Jena Bioscience). The sample was incubated on ice for 60 minutes to facilitate complete GTP-to-GMPCPP exchange. GMPCPP-stabilized MTs were then repolymerized at 37°C for 30 minutes. 8 µL of the polymerized sample was mixed with 52 µL of warm BRB80 buffer and centrifuged at 20,000 × *g* for 20 min at 37 °C. The supernatant was removed, and the pellet was resuspended in 45 µL of warm BRB80 buffer. The tubulin concentration was determined by depolymerizing MTs using 0.1 M CaCl₂ on ice for 10 min. Finally, GMPCPP-stabilized MTs were aliquoted into 5 µL portions, flash-frozen in liquid nitrogen, and stored at -80 °C.

MT preparation for pelleting assays: Tubulin aliquots were thawed and centrifuged at 10,000 × g for 10 min to remove aggregates, and the supernatant was collected for further use. GMPCPP-stabilized MTs were polymerized in cold BRB80 containing 40 μM tubulin, 1.25 mM GMPCPP, 10% glycerol, 1 mM MgCl₂ and 1 mM DTT, followed by incubation at 37 °C for 1 h. Polymerized MTs were pelleted at 10,000g for 15 min, resuspended in prewarmed BRB80, and kept at room temperature until use.

MT preparation for TIRF assay with mutant mouse tubulin: GMPCPP-stabilized MTs were polymerized from desired tubulin (WT or PTM variant tubulin) by incubation 4 mg/ml of tubulin for 30 min at 37 °C in BRB80 (80mM PIPES, 1mM EGTA, 1mM MgCl_2_, pH 6.9) supplemented with 1 mM MgCl_2_ and 1 mM GMPCPP (Jena bioscience). Polymerized MTs were diluted in BRB80 and pelleted at 18000 x g in a Microfuge 18 Centrifuge (Beckman Coulter). After centrifugation, MTs were resuspended in fresh BRB80 and kept at room temperature until usage.

**MT subtilisin treatment**

Subtilisin-treated MTs were prepared by incubating 20 μM GMPCPP-stabilized MTs with 100 μg ml⁻¹ subtilisin (Sigma-Aldrich) at 37 °C for 10 or 120 min. Reactions were terminated by addition of 2 mM PMSF. Cleaved MTs were then pelleted at 10,000 × g for 10 min at 25 °C and resuspended in 20 μL prewarmed BRB80 buffer.

**MT pelleting assay and** **statistics analysis**

Purified SkaC and Ndc80C were buffer‑exchanged into BRB80 supplemented with 50 mM KCl and 10% glycerol using Micro Spin Desalting Columns (ThermoFisher). Pelleting assays were performed using 2 μM MTs mixed with 200 nM SkaC, with or without 200 nM Ndc80C, in 20 μL reaction volumes. After a 10 min incubation at room temperature, samples were centrifuged at 100,000 × g for 10 min at 25 °C. 15 µL of the supernatant was removed from the side opposite the pellet, mixed with 4× Laemmli sample buffer (Bio‑Rad ) and boiled for 5 min. Pellets were washed once by gently adding 50 µL BRB80, discarding the wash, and resuspending the pellet in 20 µL ice‑cold BRB80 containing 50 mM CaCl_2_. After 10 min on ice, 15 µL of the suspension was mixed with 4× sample buffer and boiled for 5 min, followed by SDS-PAGE analysis.

Gels were fixed in 40% (v/v) methanol and 10% (v/v) acetic acid for 2 h, stained with Flamingo fluorescent dye (Bio‑Rad) for 3 h, briefly rinsed in 0.1 % (w/v) Tween‑20 for 10 min to reduce background and imaged on a ChemiDoc MP system (Bio‑Rad). Band intensities were quantified using Image Lab (Bio‑Rad). MT-bound proteins co-sediment with MTs and appear in the pellet, while unbound proteins stay in the supernatant. SkaC binding was quantified using the SKA2 band, and Ndc80C binding was quantified using the NDC80 band, as these bands were well resolved from the other subunits and allowed reliable densitometric measurement. The bound fraction was calculated from densitometric intensities as pellet / (pellet + supernatant). Under our assay conditions, SkaC or Ndc80C alone did not sediment in the absence of MTs. Data are mean ± SD from three independent experiments. Statistical significance was assessed with two‑tailed t‑test.

Because HeLa and porcine brain tubulin showed different polymerization efficiencies under identical assembly conditions, with HeLa tubulin consistently yielding a higher tubulin pellet fraction than porcine brain tubulin. To account for differences in the amount of assembled MTs present in each reaction, SkaC binding was normalized to the MT pellet fraction. Specifically, the fraction of SkaC recovered in the pellet was calculated as SkaC_pellet_ / (SkaC_pellet_ + SkaC_supernatant_), and this value was divided by the fraction of tubulin recovered in the pellet, Tubulin_pellet_ / (Tubulin_pellet_ + Tubulin_supernatant_), to obtain the MT-normalized SkaC fraction bound. MT-normalized Ndc80C fraction bound was calculated in the same way. Data are mean ± SD from three independent experiments. Statistical significance was assessed with two‑tailed t‑test.

**Cryo-EM grid preparation and data acquisition**

To prepare SkaC-Ndc80C-MT samples for cryo-EM analysis, a frozen aliquot of GMPCPP-stabilized MTs was thawed at 37°C for 15 minutes and diluted to a final concentration of 2.5 µM. A 4 µL aliquot of MTs was applied to a glow-discharged holey carbon grid (QuantiFoil Au R 1.2/1.3, 200 or 300 mesh) and incubated for 1 minute. After manual blotting with Whatman #1 filter paper, 3 µL of SkaC was added, followed by 1 µL of Ndc80C, which was incubated for 1 minute. The grid was then transferred to a Vitrobot (ThermoFisher) set at 25°C and 100% humidity. Blotting was performed with a force of 5 pN and a blot time of 7 seconds before plunge-freezing in liquid ethane. Samples were stored in liquid nitrogen for later use. Cryo-EM data were collected using a Titan Krios G3i microscope (ThermoFisher) operating at 300 kV and equipped with a BIO Quantum Energy Filter (Gatan). Images were recorded on a K3 direct electron detector (Gatan) at a nominal magnification of 81,000×, corresponding to a calibrated physical pixel size of 1.048 Å. Data were acquired in super-resolution mode with a dose rate of 8.003 e⁻/px/s on the detector. Each exposure lasted ~7.4 seconds and was dose-fractionated into 50 frames, corresponding to a total dose of ~50 e⁻/Å² on the specimen. Data collection was performed semi-automatically using the SerialEM software suite^77^ and parameters are summarized in Table S1.

Assembly of MT-Ndc80C-only was performed under identical conditions, except that the 2ul of Ndc80C added directly to MT, without SkaC. Cryo-EM data were collected using an Arctica microscope (ThermoFisher) operating at 200 kV. Images were recorded on a K3 direct electron detector (Gatan) at a nominal magnification of 36,000×, corresponding to a calibrated physical pixel size of 1.14 Å. The camera was operated in super-resolution mode at a dose rate of ~7.2 electrons/pixel/s on the detector. An exposure time of ~9 s was used, dose-fractionated into 50 frames, corresponding to a total accumulated dose of ~50 electrons/Å² on the specimen. Data collection was performed semi-automatically using the SerialEM software suite^77^.

**Cryo-EM data processing**

MT-Ndc80C-SkaC data processing: A total of 17,066 movie stacks were motion-corrected and dose-weighted using the RELION 3.0’s built-in implementation^78,79^. The contrast transfer function (CTF) parameters for each micrograph were estimated using Ctffind4^80^. As previously described^81,82^. MTs were manually picked in RELION 3.0. MT segments were extracted along the length of each MT using a box size of 512 pixels, with neighboring boxes spaced 82 Å apart. The PF number of each MT was determined through supervised 3D classification, using low-pass-filtered (20 Å) references of 13- and 14-pf MTs as initial models. A total of 92% of GMPCPP-MTs were classified as 14-pf MTs and selected for further processing. Global alignment parameters were determined in RELION 3.0 using a low-pass-filtered 14-pf MT map as the initial reference. The resulting alignment parameters were then imported into FREALIGN for seam refinement and further processing^83^, following our previously published protocols^84^. Refined alignment parameters from FREALIGN were subsequently imported into RELION 3.0 for symmetry expansion, after which all further processing steps were performed using RELION 5.0.

A sub-region covering 3 longitudinal and 4 lateral tubulin dimers was re-extracted with a smaller box size of 256 pixels,^85^ yielding 5,872,504 particles after symmetry expansion. Signal-subtracted datasets targeting (i) a single dimer, (ii) two longitudinal dimers, or (iii) two lateral dimers were then subjected to focused 3D classification to resolve Ndc80C-SkaC binding modes:

(i) Single dimer: Three major classes were identified: Ndc80C bound to the tubulin monomer repeat, SkaC bound to the tubulin interdimer repeat, and a poorly bound or unbound class. The Ndc80C-bound class, comprising 1,842,582 particles, was selected as the starting point for Part III processing.

(ii) Two longitudinal dimers: The classification yielded three major classes, similar to those observed for the single dimer.

(iii) Two lateral dimers: Focused 3D classification revealed three major classes: Ndc80C bound to two lateral tubulin dimers, two poorly or non-decorated lateral tubulin dimers, and SkaC and Ndc80C co-bound to two lateral tubulin dimers. The SkaC and Ndc80C co-bound class, containing 957,949 particles, was used as the starting point for Part I processing (see Part I processing details below).

Part I Tubulin-Ndc80C-SkaC complex: A total of 957,949 particles from the SkaC and Ndc80C co-bound class were selected after two rounds of lateral tubulin dimer classification. These particles were re-extracted and subjected to one round of local refinement. Next, we generated a soft mask encompassing the two lateral tubulin dimers and their associated Ndc80C and SkaC densities, which was applied for particle signal subtraction. The subtracted particles were then subjected to focused 3D classification without alignment, with Blush Regularization enabled (K = 4). A small class comprising 17.9% of particles, containing only SkaC-bound complexes, was removed. The remaining particles were reverted to their original coordinates and subjected to another round of local refinement. A second round of focused 3D classification without alignment was then performed using smaller subtracted particles, and a minor class (12.4%) containing only Ndc80C-bound complexes was excluded. This resulted in a clean dataset of SkaC and Ndc80C co-bound particles. Using the same strategy, a few more cycles of local refinement and focused 3D classification with progressively smaller masks were carried out, leading to improved visualization of structural features, such as the α-CTT. Final local refinement including 154,557 particles yielded a 2.8 Å density map of the SkaC-Ndc80C co-bound state. The final cryo-EM map was sharpened using DeepEMhancer^86^.

Part II	β-CTT: To improve the density of the β-CTT, we used the pool of 957,949 particles from the SkaC and Ndc80C co-bound class (used as a starting point for Part I). Focused 3D classification was performed using subtracted particles containing a single tubulin dimer, and 29.7% of particles with poor or lacking density for Ndc80C were removed. The remaining particles were reverted to their original coordinates and subjected to one round of local refinement. Next, a mask covering only the β- CTT tail and its surrounding region was generated, and particle subtraction was performed using this mask. Another round of focused 3D classification with subtracted particles was carried out, and a class representing 49.2% of particles with clear tail density was selected and reverted. After an additional local refinement with these reverted particles, a final round of focused 3D classification around the β- CTT region yielded a class with well-resolved β- CTT density. A total of 176,590 particles were selected for the final reconstruction, resulting in a map with an overall resolution of 3.4 Å. The final cryo-EM map was post-processed using DeepEMhancer for sharpening.

Part III Two adjacent Ndc80C coiled coils: To improve density in the coiled-coil and kink regions, we began with 1,842,582 particles from the one-tubulin-dimer classification. Two adjacent Ndc80C densities (without tubulin signal) were subtracted, followed by one round of focused 3D classification on the subtracted particles. The best class showing clear density for both α- and β-Ndc80C was selected, reverted to original coordinates, and subjected to one round of local refinement. We then re-centered and re-extracted these particles, followed by another round of local refinement. Next, two adjacent Ndc80C densities were subtracted again, and a further round of focused 3D classification was performed. A class comprising 37.8% of particles with improved coiled-coil density was selected, and 194,299 subtracted particles were refined to yield a density map containing two adjacent Ndc80 molecules at an overall resolution of 3.3 Å. The resulting map was sharpened in DeepEMhancer.

Part IV Ndc80C kink (with SKA3): For better density at the kink, we continued with the 194,299 subtracted particles from the last step of Part III. Particle subtraction was performed separately on the α- and β-Ndc80C, then combined, yielding 388,598 single Ndc80C particles for local refinement. A mask was created around the kink region, and focused 3D classification was carried out (K=3, T=80). A class comprising 34.0% of particles with a distinct helix under the kink was identified; these 115,893 particles were selected for a final round of local refinement. The resulting map was sharpened in RELION 5.0.

Part V α2-CTT: We started with 957,949 particles from the Ndc80C and SkaC co-bound class (same set used as a starting point in Parts I and II). These particles were re-extracted and recentered on the α2-tubulin tail, followed by one round of local refinement. Next, particle subtraction was performed using a mask covering the two lateral tubulin dimers, and particles from two classes displaying Ndc80C density on the right PF were selected and reverted. These reverted particles were then subjected to another round of local refinement. We then performed an additional round of particle subtraction and focused 3D classification, where 37.8% of particles displayed clear density extending from the α2-CTT to the Ndc80C on the right PF. After reversion, a reconstruction map was obtained at an overall resolution of 3.0 Å using 247,515 particles.

MT-Ndc80C-only data processing: 990 movie stacks were collected and processed using the same procedure for motion correction and CTF estimation. MT segments were extracted along the length of each filament after manual picking, using a box size of 1024 pixels with adjacent boxes spaced 82 Å apart. A total of 32,866 particles were then subjected to 2D classification in RELION 3.0.

**Cryo-EM model building, refinement and validation**

Tubulin dimers from a previously published structure (PDB: 6DPU)^87^ were rigid-body fitted into the cryo-EM density map using UCSF Chimera or Chimera X^88,89^. Because the C-terminal tail regions are less well-resolved and contain residues that vary among tubulin isoforms, they were modeled as alanine residues, except for a few positions that are 100% conserved. For the SkaC, a complete dimeric structure was predicted using AF3^60^, and the MTBD from this prediction was manually placed within the density as an initial fitting step. For the Ndc80C, relevant fragments were similarly generated by AF3 and docked into the density map.

Following these initial placements, all models were manually adjusted in Coot to correct local discrepancies with the density^90^, and further interactive model refinement was performed using ISOLDE^91^. Final real-space refinement was carried out in Phenix to optimize model geometry and ensure optimal fit to the experimental map^92^. Maps were sharpened using postprocessing in RELION and DeepEMhancer.  Local, sharpened maps were then fitted to the consensus map and merged into a single composite map of Tubulin-Ndc80-Ska using the “vop maximum” command in ChimeraX. Both the RELION and DeepEMhancer maps were used for model building, whereas only the RELION map was used for model refinement. All validation and refinement statistics are summarized in Supplementary Table 1.

**Preparation of GMPCPP-stabilized MT seeds**

Dynamic MT assays were performed using MT seeds stabilized with a slowly hydrolysable GTP analogue, GMP-CPP. The ultracentrifuge, rotor, tubes, and BRB80 buffer (80 mM PIPES, 1 mM MgCl_2_, 1 mM EGTA, 1 mM DTT, pH 6.8 adjusted with KOH) were prechilled. Tubulin was mixed with 5% biotin-tubulin and 7% LD655-labeled tubulin, diluted to 15 mg mL^-1^ in cold BRB80, and incubated on ice for 10 min. The mixture was cold spun at 400,000 *g* for 10 min to remove inactive tubulin. The supernatant was transferred to a fresh tube and GMP-CPP was added to a final concentration of 1mM followed by addition of DMSO to a final concentration of 10% and incubated at 37 °C for 30 min. After equilibrating the centrifuge and buffer to 37 °C, the sample was spun again at 400,000 *g* for 10 min. The pellet was gently resuspended in 30 µL of warm BRB80 using a cut pipette tip to prevent shearing. Aliquots of GMPCPP-stabilized seeds were snap-frozen and stored at -80 °C.

**Dynamic microtubule imaging**

Tubulin was clarified by ultracentrifugation at 400,000 g for 10 min at 4 °C. Biotinylated GMPCPP-stabilized seeds were immobilized on biotin-PEG-coated coverslips via streptavidin linkage. Imaging chambers were filled with a dynamic MT mixture (1 mg mL-1 casein, 0.5% Pluronic F-127, 0.5 mM DTT, 2 mM GTP, 0.2% methylcellulose, 0.1 mg/mL glucose oxidase, 0.2 mg/mL catalase, 0.8% D-glucose in BRB80 pH 6.8) containing 100nM of SkaC (or its mutants) and/ or 50nM of Ndc80C-eGFP and tubulin at a concentration of 1.5mg/ml (which had 7% of labelled cy3 tubulin) and was then sealed with nail polish to prevent evaporation during the course of imaging. Imaging was performed at 33 °C. Images were captured every 5 s for 150 frames per imaging area and analyzed as kymographs made in FIJI. For the MT lattice selection of Ndc80C-eGFP and SkaC-6D-mCherry, unlabeled tubulin was used except for the SkaC-6D-mCherry only condition where 7% of 488 labelled tubulin was used.

**Measurement of dissociation kinetics of SkaC-6D-mCherry and Ndc80C-eGFP**

Flow chambers were assembled by sandwiching a glass slide (drilled with two holes) and biotinylated coverslip using a permanent double-sided tape. GMPCPP stabilized MTs were immobilized on the biotin-PEG-coated surface via streptavidin linkage. Imaging chambers were then filled imaging buffer (1 mg mL-1 casein, 0.5% Pluronic F-127, 0.5 mM DTT, 0.2% methylcellulose, 0.1 mg/mL glucose oxidase, 0.2 mg/mL catalase, 0.8% D-glucose in BRB80 pH 6.8) containing SkaC-6D-mCherry (180 nM when alone or 100 nM when in presence of Ndc80C-eGFP) and/or 50nM Ndc80C-eGFP. Imaging was initiated and after acquisition of ~15 frames the chamber was washed with imaging buffer during continued image acquisition to monitor the bulk detachment of the proteins from the GMPCPP MTs.

**Tip tracking**

To test tip tracking we used LD655 labelled SkaC-6D_FL-SNAP and SkaC-6D_DEL-SNAP along with Ndc80C-eGFP. Experiments were performed in flow chambers that were assembled with glass slide containing two drilled holes as described above. Dynamic MTs were polymerized from immobilized GMPCPP seeds, as described above, at 33 °C in dynamic MT buffer (1 mg mL-1 casein, 0.5% Pluronic F-127, 0.5 mM DTT, 2 mM GTP, 0.2% methylcellulose, 0.1 mg/mL glucose oxidase, 0.2 mg/mL catalase, 0.8% D-glucose in BRB80 pH 6.8) containing tubulin at a concentration of 1.5mg/ml. Depolymerization of MT was triggered by removal of soluble tubulin with imaging buffer (1 mg mL-1 casein, 0.5% Pluronic F-127, 0.5 mM DTT, 0.2% methylcellulose, 0.1 mg/mL glucose oxidase, 0.2 mg/mL catalase, 0.8% D-glucose in BRB80 pH 6.8) containing the indicated protein for visualization of tip-tracking. SkaC-6D_FL-SNAP and SkaC-6D_DEL-SNAP were used at a concentration of 25 nM and Ndc80C-eGFP was used at a concentration of 30 nM. Images were captured every 200 ms and analyzed as kymograph in FIJI.

**TIRF Microscopy**

For the dynamic MT assay, total internal reflection fluorescence (TIRF) microscopy was performed on a custom-built multicolor objective-type TIRF microscope based on a Nikon Ti-E body equipped with a 100×, 1.49 N.A. oil-immersion apochromatic objective and Perfect Focus System. Fluorescence was detected using an electron-multiplying CCD camera (Ixon EM+, Andor; 512 × 512 pixels) with an effective pixel size of 160 nm after magnification. eGFP, Cy3 or mCherry, and LD655 fluorophores were excited with 488, 561, and 633 nm lasers (Coherent) coupled through a single-mode fiber (Oz Optics), and emission was collected through 525/50, 593/46 (Cy3) or or 629/56 (mCherry), and 697/75 nm bandpass filters (Semrock), respectively. The system was controlled with Micro-Manager v1.4. For the TIRF assays using mutant mouse tubulin, TIRF microscopy assays were performed on an inverted widefield microscope (Nikon Eclipse Ti2), equipped with motorized XY-stage, transmitted light lamp (Nikon TI2-D-LHLED), Nikon Apo TIRF 60x oil immersion objective (NA 1.49), 1.5x tube lens, and PRIME BSI camera (Teledyne Photometrics). MTs were visualized using interference reflection microscopy (IRM) and Ndc80-eGFP and Ska-mCherry were imaged by using an EM435-485 and EM500-545 filter turrets, respectively.

**TIRF microscopy with mouse mutant and wild-type brain tubulin**

TIRF chambers were assembled by melting thin parafilm strips in between two glass coverslips silanized with Hexamethyldisilazane (HMDS, Sigma, #379212). Chambers were incubated with 20 µg/mL anti-β-tubulin antibodies (#T7816, Sigma) and passivated with 1% F-127 (#P2443, Sigma, 1% in PBS). The PTM-variant MTs were immobilized into the chamber, identified using IRM, after which WT MTs were immobilized. Before imaging, chambers were equilibrated in TIRF assay buffer AB (BRB80 pH 6.9, supplemented with 10 mM dithiothreitol, 0.02 mg/ml casein, 1 mM Mg-ATP, 20 mM D-glucose, 0.1% Tween20, 0.22 mg/ml glucose oxidase and 20 μg/ml catalase). Next, Ndc80C-eGFP and SkaC-mCherry were diluted to 4 nM in AB, added to the chamber, and imaged using TIRF microscopy.

**Data analysis for TIRF microscopy**

Dynamic MT was captured for 150 frames per imaging area and analyzed as kymographs made in FIJI. Catastrophe frequency was determined by counting the number of plus-end catastrophe events on each MT and then dividing this number by the total data collection time. Rescue fraction was determined by counting the number of plus-end rescue events on each MT and then dividing this number by the number of catastrophes on the same MT. Background-subtracted integrated fluorescence intensity of Ndc80C-eGFPa and SkaC-6d-mCherry on the GMPCPP or GDP MT was calculated in FIJI.

For the off-rate measurement experiment, the integrated fluorescence intensity was calculated from each MT and photo bleach corrected using a custom-written macro in FIJI. The integrated fluorescence intensity was normalized from each MT fitted using a bi-exponential function and the rate constants were calculated as the weighted average of the two rates, using custom-written code in MATLAB.

The P values for the two-tailed Student’s t-test were calculated in GraphPad Prism.

In the mouse mutant tubulin and concentration dependency assay, Ndc80C-eGFP or SkaC-mCherry density on the different MT variants was measured after 5 minutes of incubation and quantified using ImageJ2 (2.16.0/1.54g). For each experiment, Ndc80C or SkaC density was measured as the mean intensity of a 5-pixel-wide line drawn along the microtubule in the 488- or 561- channel, respectively. Background was estimated for each MT by drawing an identical line directly adjacent to the MT, and the background mean was subtracted from the MT mean. For the mouse mutant tubulin assay, the Ndc80C or SkaC density on the PTM-variant MTs was then normalized to the average density on the WT MTs of the same experiment.

**Extended Data Fig. 1: Purification and characterization of the SkaC-6D and Ndc80C samples, used for cryo-EM analyses.** **a,** Schematic of the human SkaC-6D construct, with phosphomimetic substitutions in SKA3 at T203, T217, S283, T291, T358, and T360. Regions resolved in the cryo-EM maps are shaded (SKA1: 136-255; SKA3: 357-380). **b,** SDS-PAGE gel of the SkaC-6D after gel filtration. Bands corresponding to the three SkaC subunits are indicated. **c,** Mass photometry analysis of SkaC-6D sample showing a major species at the expected molecular mass for the SkaC dimeric complex. The measured molecular masses, associated errors, and relative populations are indicated. **d,** Schematic of the human Ndc80C construct. Regions resolved in the cryo-EM maps are shaded (NDC80: 16-24, 68-327; NUF2: 1-216). **e,** SDS-PAGE gel of the Ndc80C sample after gel filtration. Bands corresponding to the four Ndc80C subunits are labeled. **f,** Mass photometry analysis of Ndc80C showing a major species at the expected molecular mass for the Ndc80 complex. Measured molecular masses, corresponding errors, and population percentages are indicated.

**Extended Data Fig. 2: Data processing workflow for the overall MT-Ndc80C-SkaC and Tubulin-Ndc80C-SkaC subregions****.**

**a,** Representative cryo-EM micrograph of the MT-Ndc80C-only sample. The MT density is highlighted in yellow (same for (**b**)). **b,** Representative cryo-EM micrograph of the MT-Ndc80C-SkaC sample. **c,** Data processing flowchart for the initial reconstruction of the MT-Ndc80C-SkaC. **d,** Focused 3D classification using masks encompassing a single tubulin dimer (left), two longitudinal tubulin dimers (middle), or two lateral tubulin dimers (right). **e,** Detailed processing flowchart for the Tubulin-Ndc80C-SkaC sub-region (Part I) analysis.

**Extended Data Fig. 3: Data processing workflow of β-tubulin tail, Ndc80C coiled-coil, Ndc80C kink-SKA3 and α2-tubulin tail subregion analyses. a,** Data processing flowchart for the β-tubulin tail sub-region (Part II). **b,** Data processing flowchart of two adjacent Ndc80C coiled coils (Part III) and the Ndc80C kink region with SKA3 (Part IV). **c,** Data processing flowchart for the α2-tubulin tail sub-region (Part V).

**Extended Data Fig. 4: Map quality analysis for each reconstruction. a,** Composite map of the Tubulin-Ndc80C-SkaC. Dashed boxes indicate regions subjected to local refinement during processing. The map clearly distinguishes the s9-s10 loop differences between α- and β-tubulin. **b,** Local-resolution maps and corresponding FSC curves for each locally refined subregion: the consensus Tubulin-Ndc80C-SkaC map (Part I) and focused maps of β-tubulin (Part II), two adjacent Ndc80Cs (Part III), and the Ndc80C kink region and SKA3 (Par IV).

**Extended Data Fig. 5: Composite, average map and tubulin-tail analyses of Ndc80C and SkaC binding on the MT lattice. a,** Composite averaged cryo-EM density map of Ndc80C and SkaC bound to the MT. The dashed box marks three PFs, each containing two longitudinal tubulin dimers. **b,** Enlarged views of the boxed region in (**a**). In the averaged map, Ndc80C and SkaC densities overlap on the MT surface (asterisk, yellow dashed circle). **c,** Alignment of α-tubulin tail sequences from the indicated isoforms. **d,** Surface representation of the cryo-EM map showing the α2-tubulin tail extending toward the NDC80 N-tail on the adjacent PF. **e,** Surface representation of the cryo-EM map (left) and transparent map with the fitted atomic model (right) showing β-tubulin tail density resolved to E435 in the presence of Ndc80C. **f,** SDS-PAGE analysis of MT pelleting assays showing Ndc80C and SkaC-6D binding to untreated MTs or to MTs treated with subtilisin for 10 min or 120 min, as indicated. Pellet (P) and supernatant (S) fractions are shown. From left to right, sample groups correspond to Ndc80C alone, SkaC-6D alone, and Ndc80C plus SkaC-6D.

**Extended Data Fig. 6: Sequence alignments between Hela and Porcine tubulin.** Sequence alignments of α- (**a**) and β-tubulin (**b**) from HeLa cells and porcine brain tubulin, using the major isotype in each case (human α-tubulin: TUBA1B, UniProt P68363, and TUBA1C, UniProt Q9BQE3; human β-tubulin: TUBB/TUBB5, UniProt P07437; porcine α-tubulin: TUBA1B, UniProt Q2XVP4; porcine β-tubulin: UniProt P02554). Sequence alignments were generated with ClustalW^93^ and displayed using ESPRIPT^94^ notation, with identical residues highlighted in red and similar residues shown in red text boxed in blue. The same notation is used throughout all sequence alignments in this study.

**Extended Data Fig. 7:** **Concentration dependency of Ndc80C and SkaC on WT brain and HeLa MTs. a,** Density of Ndc80C on GMPCPP MTs assembled from wild-type (WT) mouse brain (red) or HeLa (blue) tubulin in presence (solid lines) or absence (dotted lines) of SkaC. Data are presented as mean ± SD (n=32, 39, 43, 47 MTs, respectively in 1 independent experiment for each condition). **b,** Density of SkaC on GMPCPP MTs assembled from wild-type (WT) mouse brain (red) or HeLa (blue) tubulin in presence (solid lines) or absence (dotted lines) of Ndc80C. Data are presented as mean ± SD (n=31, 39, 36, 36 MTs, respectively in 1 independent experiment for each condition).

**Extended Data Fig. 8: Structural and sequence conservation of the Ndc80C-MT interface. a,b,** Cryo-EM density fitted with the atomic model showing the interface between α-Ndc80C and α-tubulin. Key residues are labeled, and putative salt bridges and hydrogen bonds are indicated by black dashed lines (same for (**d**)). DeepEMhancer sharpened maps are used for visualization (same for (**d**)). **c,** Hydrophobic contacts in (**b)**, with α-tubulin shown as a surface colored by the Kyte-Doolittle scale (brown, hydrophobic; green, hydrophilic; white, intermediate). α-Ndc80C shown as cartoon. The same coloring scheme is used for all hydrophobic-interaction panels. **d,** Cryo-EM map fitted with the atomic model showing the interface between α-NDC80 and β-tubulin. **e,f,** Sequence alignments of porcine α- and β-tubulin regions that contribute to the Ndc80C-binding interface. α-tubulin is used as the reference in (**e**), and β-tubulin is used as the reference in (**f**). Arrows mark residues involved in Ndc80C contacts. Several interface residues are conserved between α- and β-tubulin, while others show conservative acidic, basic, or hydrophobic/aromatic substitutions. The major porcine brain tubulin isotypes were used.

**Extended Data Fig. 9: Structural and evolutionary features of a putative cooperative interface between neighboring Ndc80 complexes.** **a,** Cryo-EM map fitted with model showing the interface between α-NDC80 and β-NDC80. **b,** Hydrophobic contacts in (**a**). **c,** Electrostatic surface of the α-Ndc80C and cartoon of β-Ndc80C showing NDC80 S69 facing an acidic surface in the former. **d,** Sequence alignment of NDC80 residues 66-118 and NUF2 residues 59-110. Residues contributing to cooperative binding are indicated by black arrows. Residue K115 in NDC80 is largely conserved across species, except in Drosophila and C. elegans. In contrast, NDC80 residues F74 and E78, as well as NUF2 Y65 and E80, are highly conserved but only among vertebrates. By comparison, NDC80 A109/H110 and NUF2 L61 show limited conservation within vertebrates. These conservation patterns indicate that the cooperative binding interface is selectively reinforced in vertebrates. Sequence labels denote UniProt accession and species: HUMAN (*Homo sapiens*); CHICK (*Gallus gallus*); XENLA (*Xenopus laevis*); DANRE (*Danio rerio*); ARATH (*Arabidopsis thaliana*); DROME (*Drosophila melanogaster*); CAEEL (*Caenorhabditis elegans*); YEAST (*Saccharomyces cerevisiae*).

**Extended Data Fig. 10: Additional regions of the Ndc80C coiled-coil that may contribute to cooperativity, and the composite full-length model of human Ndc80C. a,** Cryo-EM map showing two Ndc80C bound to a single tubulin dimer. The black dashed box highlights the region of possible contact between adjacent Ndc80C at the kink; the red dashed box indicates an additional region between adjacent tails. The kink, canonical hinge, and terminal residues of our model are listed and marked by black arrows. **b,** Superposition of human (this study) and yeast NDC80 and NUF2 (EMDB: 18304) cryo-EM maps highlights a kink in the human Ndc80C coiled-coil region that is absent in yeast (beige). **c,** AF3-predicted model of three Ndc80C coiled-coil regions (NDC80^367-642^/NUF2^250-464^) arranged as a bundle with ~40 Å spacing between adjacent coiled-coils (upper panel), colored based on confidence of the prediction. For clarity, the lower panel shows only two AF3 models, with residues contributing to intermolecular contacts highlighted in red and green. The dashed box marks the loop region. **d,** A representative 2D class average from the MT-Ndc80C-only sample. The composite full-length of human Ndc80C model using experimental section and AF3 for the rest of the coiled-coil is overall consistent with the observed density in 2D average. Kink, canonical hinge and loop regions are indicated by white arrows for the first complex on the top right.

**Extended Data Fig. 11: Structural basis of SKA1-MTBD binding to the MT lattice and comparison with XL-MS interaction sites.** **a**-**d,** Cryo-EM density fitted with atomic models showing the interface between SKA1-MTBD and α1-tubulin (**a**), α2-tubulin (**b**-**c**), and β2-tubulin (**d**). Key residues are labeled and putative salt bridges and hydrogen bonds are indicated by black dashed lines. DeepEMhancer sharpened maps are used for visualization (same for (**a**-**d**)). **e**-**f,** Hydrophobic contacts between SKA1 and α2-tubulin (**e**) or β2-tubulin (**f**), with SKA1 shown as a cartoon and tubulin rendered as hydrophobic/hydrophilic surfaces using the Kyte-Doolittle coloring. **g,** Previously published XL-MS data^33^ identifying interaction patches between SKA1-MTBD and tubulin, reproduced from Abad et al. (2014) under a CC BY 3.0 license. **h,i,** For XL-MS patches involving SKA1 K183/K184 (**h**) or K203/K206 (**i**), the corresponding positions in our structure are separated by more than 30 Å, suggesting that these contacts are not captured by the straight-lattice interface resolved here and may instead arise from interactions with curved tubulin conformations.

**Extended Data Fig. 12:** **Comparison of the AF3-predicted structure with our cryo-EM based model for the Ndc80C coiled-coil kink region interacting with SKA3. a,** AF3 predicted structure for NDC80^223-360^/NUF2^116-245^/SKA3-6D^355-380^ colored based on model confidence. The corresponding predicted aligned error (PAE) plot is shown on the right. **b,** All five AF3 models from panel (**a**) are superimposed on the SKA3 tether helix and show differences in the angle at the kink. The red dashed box highlights the conserved interaction interface common to all models. **c,** Superposition of our experimentally-based model (colored as in the main figures) with the highest-confidence AF3 model (shown in green) shows a similar interaction interface but a distinct Ndc80C kink angle.

**Supplemental Data Table 1. Cryo-EM data collection, refinement and validation statistics**

|  | #1 Composite map and model of Tubulin-Ndc80-Ska (EMDB-75942) (PDB-11QA) | #2 Consensus map of Tubulin-Ndc80-Ska  (EMD-75938) | #3 Beta-tubulin  (EMD-75939) | #4 Two adjacent Ndc80s (EMD-75940) | #5 Ndc80 kink and SKA3  (EMDB-75941) |
| --- | --- | --- | --- | --- | --- |
| **Data collection and processing** |  |  |  |  |  |
| Microscope |  | Titan  Krios | Titan Krios | Titan Krios | Titan Krios |
| Magnification |  | 81,000 | 81,000 | 81,000 | 81,000 |
| Voltage (kV) |  | 300 | 300 | 300 | 300 |
| Detector |  | Gatan K3 | Gatan K3 | Gatan K3 | Gatan K3 |
| Energy filter slit width (eV) |  | 20 | 20 | 20 | 20 |
| Electron exposure (e–/Å^2^) |  | 50 | 50 | 50 | 50 |
| Defocus range (μm) |  | -0.7 to -1.5 | -0.7 to -1.5 | -0.7 to -1.5 | -0.7 to -1.5 |
| Pixel size (Å) |  | 1.048 | 1.048 | 1.048 | 1.048 |
| Symmetry imposed |  | C1 | C1 | C1 | C1 |
| Micrographs collected (no.) |  | 17,066 | 17,066 | 17,066 | 17,066 |
| Initial particle images (no.) |  | 5,872,504 | 5,872,504 | 5,872,504 | 5,872,504 |
| Final particle images (no.) |  | 154,557 | 176,590 | 194,299 | 115,893 |
| Map resolution (Å)  FSC threshold |  | 2.8  0.143 | 3.4  0.143 | 3.3  0.143 | 4.7  0.143 |
|  |  |  |  |  |  |
| **Refinement** |  |  |  |  |  |
| Initial model used (PDB code) | 6DPU, AlphaFold3 |  |  |  |  |
| Refinement packages | Phenix/Coot/Isolde |  |  |  |  |
| Model resolution (Å)  FSC threshold  Model-Map scores | 3.3  0.5  0.79 |  |  |  |  |
| Model composition  Non-hydrogen atoms  Protein residues  Ligands | 22745  2863  8 (2GTP 2G2P 4MG) |  |  |  |  |
| *B* factors (Å^2^)  Protein  Ligand | 87.27  64.67 |  |  |  |  |
| R.m.s. deviations  Bond lengths (Å)  Bond angles (°) | 0.005  0.523 |  |  |  |  |
| Validation  MolProbity score  Clashscore  Rotamers outliers (%)  Cβ deviations (%)  CaBLAM outliers (%) | 2.10  7.47  3.39  N/A  2.07 |  |  |  |  |
| Ramachandran plot  Favored (%)  Allowed (%)  Outliers (%) | 95.91  3.99  0.11 |  |  |  |  |
